# SARS-CoV-2 antibody dynamics in blood donors and COVID-19 epidemiology in eight Brazilian state capitals: A serial cross-sectional study

**DOI:** 10.7554/eLife.78233

**Published:** 2022-09-22

**Authors:** Carlos A Prete, Lewis F Buss, Charles Whittaker, Tassila Salomon, Marcio K Oikawa, Rafael HM Pereira, Isabel CG Moura, Lucas Delerino, Manoel Barral-Netto, Natalia M Tavares, Rafael FO Franca, Viviane S Boaventura, Fabio Miyajima, Alfredo Mendrone-Junior, Cesar de Almeida-Neto, Nanci A Salles, Suzete C Ferreira, Karine A Fladzinski, Luana M de Souza, Luciane K Schier, Patricia M Inoue, Lilyane A Xabregas, Myuki AE Crispim, Nelson Fraiji, Fernando LV Araujo, Luciana MB Carlos, Veridiana Pessoa, Maisa A Ribeiro, Rosenvaldo E de Souza, Sônia MN da Silva, Anna F Cavalcante, Maria IB Valença, Maria V da Silva, Esther Lopes, Luiz A Filho, Sheila OG Mateos, Gabrielle T Nunes, Alexander L Silva-Junior, Michael P Busch, Marcia C Castro, Christopher Dye, Oliver Ratmann, Nuno R Faria, Vítor H Nascimento, Ester C Sabino

**Affiliations:** 1 Department of Electronic Systems Engineering, University of São Paulo São Paulo Brazil; 2 Imperial College London London United Kingdom; 3 MRC Centre for Global Infectious Disease Analysis, Imperial College London London United Kingdom; 4 Abdul Latif Jameel Institute for Disease and Emergency Analytics, Imperial College London London United Kingdom; 5 Faculdade Ciências Médicas de Minas Gerais Belo Horizonte Brazil; 6 Universidade Federal do ABC São Bernardo do Campo Brazil; 7 Institute for Applied Economic Research (Ipea) Brasília Brazil; 8 Fundação Oswaldo Cruz Manguinhos Brazil; 9 Universidade Federal do Ceará Fortaleza Brazil; 10 Fundação Pró Sangue Hemocentro de São Paulo (FPS) São Paulo Brazil; 11 Centro de Hematologia e Hemoterapia do Paraná (HEMEPAR) Curitiba Brazil; 12 Fundação Hospitalar de Hematologia e Hemoterapia do Amazonas (HEMOAM) Manaus Brazil; 13 Fundação de Hematologia e Hemoterapia da Bahia (HEMOBA) Salvador Brazil; 14 Centro de Hematologia e Hemoterapia do Ceará (HEMOCE) Fortaleza Brazil; 15 Fundação HEMOMINAS Belo Horizotne Brazil; 16 Fundação de Hematologia e Hemoterapia de Pernambuco (HEMOPE) Recife Brazil; 17 Instituto Estadual de Hematologia Arthur de Siqueira Cavalcanti (HEMORIO) Rio de Janeiro Brazil; 18 Universidade Federal do Amazonas Manaus Brazil; 19 Centro Universitário do Norte Manaus Brazil; 20 Vitalant Research Institute San Franscico United States; 21 University of California, San Francisco San Francisco United States; 22 Harvard TH Chan School of Public Health Boston United States; 23 Department of Zoology, University of Oxford Oxford United Kingdom; 24 Instituto de Medicina Tropical, University of São Paulo São Paulo Brazil; The University of Melbourne Australia; University of New South Wales Australia

**Keywords:** SARS-CoV-2, COVID-19, blood donors, serosurveillance, attack rate, infection fatality rate, Viruses

## Abstract

**Background:**

The COVID-19 situation in Brazil is complex due to large differences in the shape and size of regional epidemics. Understanding these patterns is crucial to understand future outbreaks of SARS-CoV-2 or other respiratory pathogens in the country.

**Methods:**

We tested 97,950 blood donation samples for IgG antibodies from March 2020 to March 2021 in 8 of Brazil’s most populous cities. Residential postal codes were used to obtain representative samples. Weekly age- and sex-specific seroprevalence were estimated by correcting the crude seroprevalence by test sensitivity, specificity, and antibody waning.

**Results:**

The inferred attack rate of SARS-CoV-2 in December 2020, before the Gamma variant of concern (VOC) was dominant, ranged from 19.3% (95% credible interval [CrI] 17.5–21.2%) in Curitiba to 75.0% (95% CrI 70.8–80.3%) in Manaus. Seroprevalence was consistently smaller in women and donors older than 55 years. The age-specific infection fatality rate (IFR) differed between cities and consistently increased with age. The infection hospitalisation rate increased significantly during the Gamma-dominated second wave in Manaus, suggesting increased morbidity of the Gamma VOC compared to previous variants circulating in Manaus. The higher disease penetrance associated with the health system’s collapse increased the overall IFR by a minimum factor of 2.91 (95% CrI 2.43–3.53).

**Conclusions:**

These results highlight the utility of blood donor serosurveillance to track epidemic maturity and demonstrate demographic and spatial heterogeneity in SARS-CoV-2 spread.

**Funding:**

This work was supported by Itaú Unibanco ‘Todos pela Saude’ program; FAPESP (grants 18/14389-0, 2019/21585-0); Wellcome Trust and Royal Society Sir Henry Dale Fellowship 204311/Z/16/Z; the Gates Foundation (INV- 034540 and INV-034652); REDS-IV-P (grant HHSN268201100007I); the UK Medical Research Council (MR/S0195/1, MR/V038109/1); CAPES; CNPq (304714/2018-6); Fundação Faculdade de Medicina; Programa Inova Fiocruz-CE/Funcap - Edital 01/2020 Number: FIO-0167-00065.01.00/20 SPU N°06531047/2020; JBS – Fazer o bem faz bem.

## Introduction

Brazil has experienced one of the world’s most significant COVID-19 epidemics, with over 22 million cases and 621,000 deaths reported as of 14 January 2022. However, this national picture masks important sub-national heterogeneity, with extensive variation in SARS-CoV-2 spread between population groups (Li et al., 2021) and locations (Castro et al., 2021; Hallal et al., 2020) as well as regional differences in the stringency of non-pharmaceutical interventions (de Souza Santos et al., 2021).

Understanding the drivers of these differences is crucial, both retrospectively as a means of evaluating past attempts at controlling spread, and as a guide to the potential impact of future transmission. Indeed, a significant fraction of the COVID-19 burden in Brazil was driven by the emergence of the Gamma (P.1) variant of concern (VOC) in November 2020, which drove extensive resurgence of transmission following its apparent emergence in the Amazonas State capital city of Manaus. Despite the evidence of high levels of population-level immunity that should have hindered further transmission (Buss et al., 2021), a phenomenon attributed to the Gamma VOCs likely increased transmissibility and ability to partially evade immune responses (Faria et al., 2021). Subsequent spread to the rest of Brazil led to similar resurgence, extensive transmission, and disease burden leading to substantial pressure on health systems (Brizzi et al., 2021; de Oliveira et al., 2021; Martins et al., 2021). As with the first epidemic wave, the degree and extent to which different locations were affected varied markedly. Understanding the drivers of this variation is crucial to shed light on how and why SARS-CoV-2 spreads across different populations, and how past epidemics shape subsequent transmission of the virus. More generally, because previous natural infection may enhance vaccine response (Crotty, 2021; Reynolds et al., 2021; Stamatatos et al., 2021), understanding the extent of previous exposure in the country may have important implications for the development of epidemic waves driven by new variants in the context of the ongoing large-scale, nationwide vaccination campaign.

Here, we analyse the divergent epidemic SARS-CoV-2 dynamics in eight of the biggest Brazilian cities (Belo Horizonte, Curitiba, Fortaleza, Manaus, Recife, Rio de Janeiro, Salvador, and São Paulo). We estimate the seroprevalence over time for these cities disaggregated by age and sex using repeated cross-sectional convenience samples of routine blood donors collected from March 2020 to March 2021. We also provide estimates for the age-specific infection fatality rates (IFR, defined as the number of deaths per infection) and infection hospitalisation rates (IHR, the number of hospitalisations per infection) for these cities. In Manaus, the Gamma VOC became dominant before March 2021 (see Appendix 1—figure 1), enabling us to provide estimates of Gamma’s IFR and IHR. Our results highlight important differences in the drivers of SARS-CoV-2 epidemic spread across Brazil’s major population centres and underscore the utility of blood donors for regular serosurveillance as a tool to track progression of epidemics of emerging infectious diseases.

## Methods

### Selection of blood donors for estimation of seroprevalence

Each of the eight cities had a monthly quota of 1,000 kits for testing selected donation samples in this study. In order to select more representative samples, we selected blood samples so that the spatial distribution of residential location of selected donors matches the spatial distribution of population density in each municipality. More specifically, each city was divided into sub-municipal administrative zones, and the original quota (1,000 kits) was divided into sub-quotas following the populational distribution of the city administrative zones. Starting from the second week of each month, we selected consecutive blood donors based on the geolocation of their residential postcode to fill the sub-quotas. In this way, donations with missing or wrong postal code were considered ineligible for selection. We chose the sample size (1,000) so an increase in crude seroprevalence of 5% can be detected with power 1-β=80% and confidence level 1-α=95% assuming a baseline seroprevalence of 15%.

In Manaus, however, donor postcodes were not reliably collected, so that the number of missing and wrong values makes this strategy unfeasible. So, samples were selected consecutively with no postal code restrictions. We also developed a study management system to operationalize this sampling strategy, whereby blood donor postcodes and epidemiological data were automatically extracted and selected. After that, the selected donation sample IDs were released for the research assistant to be separated for testing.

From 453,211 available blood samples collected in all 8 cities except Manaus, 72,783 had a missing or invalid residential postal code, and 198,199 were from individuals living in regions not included in this study, thus 182,229 samples were eligible for selection. An average of 1010 samples were selected monthly for each city from March 2020 to March 2021, except for Recife where tests occurred until February 2021. A total of 104,013 samples were selected, but 6063 samples could not be retrieved or did not have enough volume to be tested, leading to 97,950 tested samples (951 samples per month in average for each city). Appendix 1—figure 2 contains a flowchart describing the selection procedure of blood donors.

In Brazil, blood donation samples are usually saved for 6 months, so when serological test kits were made available in July 2020, we could retrospectively select and test frozen samples from February to July. After this, period samples were selected and tested in real time. Antibody tests results were not made available to the blood donors themselves.

Blood donors are a convenience sample, and thus may not be representative of the wider population in terms of their risk of SARS-CoV-2 exposure. Appendix 1—figures 3–6 show a comparison between recorded blood donor demographics and the last available Brazilian census conducted in 2010. Donors differ systematically in age, sex, and self-reported skin colour compared to the population, but the income per capita is similar. To account for the differences in the age-sex structure of blood donors, we divide donors in age-sex groups and estimate the prevalence of each age-sex group separately. Then, we calculate the seroprevalence of the population as a weighted sum of the seroprevalences of each age-sex group.

### SARS-CoV-2 serology assays

We applied chemiluminescent microparticle immunoassays (CIMA, AdviseDx, Abbott) that detect IgG antibodies against the SARS-CoV-2 nucleocapsid (N) because it was the only automated commercially available kit in Brazil when the study started (July 2020). We used this kit throughout the study until March in all eight cities except Recife, where we used the kit until February 2021. This assay suffers from signal waning - resulting in positive-negative transition, or ‘seroreversion’ - during convalescence. This amounts to a fall in assay sensitivity through time. The Abbott anti-N IgG CMIA shows particularly rapid signal decay when compared with other assays (Di Germanio et al., 2021). These antibody dynamics mean that as an epidemic progresses, the crude proportion of individuals with a positive test result will increasingly underestimate the true attack rate (Buss et al., 2021; Takahashi et al., 2021; Takahashi et al., 2020).

A test is considered positive if the obtained signal to cutoff (S/C) is greater or equal to a predefined threshold of 0.49. This is the lower threshold recommended by the manufacturer, which was used instead of the upper threshold of 1.4 to partially attenuate the effect of seroreversion. Appendix 1—figures 7–9 contain the number of tests disaggregated by month, age, sex and the monthly S/C distribution. We also decided to validate the results observed in Manaus, as this represents a unique sentinel population, by retesting all samples in November 2020 using the CIMA (AdviseDx, Abbott) that detects IgG antibodies against the SARS-CoV-2 spike (S) protein (see Appendix 1 for the validation analyses).

To determine the test sensitivity, we considered a cohort of 208 non-hospitalised symptomatic SARS-CoV-2 PCR-positive convalescent plasma donors tested within 60 days after symptom onset (Supplementary file 1). These donors had symptomatic COVID-19 with PCR-confirmed SARS-CoV-2 infection and were recruited to provide convalescent plasma. We found a sensitivity of 90.6% for the anti-N assay using a threshold of 0.49 S/C and 94.0% for the anti-S assay. Specificity for the anti-N assay was 97.5%, with 801 negative results in 821 pre-pandemic blood donation samples (Buss et al., 2021). Sensitivity and specificity for other assay thresholds are shown in Supplementary file 1. The anti-S assay has a specificity of >99% (Di Germanio et al., 2021; Stone et al., 2021), and we assume 100% in this study. Although the sensitivity of both assays declines through time due to waning of the detected antibodies below the positivity threshold, the anti-S IgG antibodies wane more slowly (Di Germanio et al., 2021; Stone et al., 2021). Sensitivity obtained from convalescent plasma donors is likely overestimated due to spectrum bias. This is because convalescent donors had moderate-to-severe SARS-CoV-2 infection, and thus differ from the whole blood donor population (used to estimate seroprevalence), who are more likely to have had asymptomatic or mild disease.

We subsequently estimated the distribution of time to seroreversion, and thus the sensitivity decreasing through time, for the anti-N assay. We first calculated this in the convalescent donors, in whom the date of symptom onset is known, and whose blood samples were collected longitudinally during convalescence. As such, the time-to-seroreversion distribution was computed after accounting for right censoring. However, due to spectrum bias, the extrapolation of antibody waning from convalescent donors to whole blood donors is unlikely to be valid. As such, we obtained a second cohort of repeat blood donors in Manaus that provided multiple donations during the 2020–2021 period. These donors are expected to have the same antibody dynamics as the seroprevalence cohort, as they are drawn from the same population and have predominantly mild or asymptomatic infections. However, in this group the time of infection is unknown, as infection is inferred by serostatus alone. The procedure to manage this problem is described below.

### Methods used to estimate the time-dependent sensitivity

We developed an analytic method to correct raw seroprevalence data for seroreversion, improving on the method used in Buss et al., 2021. We first estimate the time-to-seroreversion distribution using serial donations from repeat blood donors, which determines how sensitivity for a given individual decreases with the time after seroconversion. We then corrected the raw seroprevalence estimates for the changing sensitivity within a Bayesian framework. We first calculated attack rates for each age and sex group in each city and summed these using the proportion of each group in the Brazilian reference population to obtain standardised estimates. In this section, we describe a procedure to estimate the time-dependent sensitivity used to obtain a seroprevalence estimate corrected for antibody waning.

Let ${se}_{0}$ be the sensitivity measured shortly after symptomatic infection (i.e. the probability of an infected individual seroconverting to an S/C above the threshold), and $p^{+}\left[n\right]$ be the probability of a donor remaining positive n weeks after seroconversion (given that the donor seroconverted). Then, the sensitivity of the test n weeks after seroconversion is $se_{0}\times p^{+}\left[n\right]$ for a given donor. In this section, we describe the procedure used to determine $p^{+}\left[n\right]$ from repeat blood donors data, for which time of infection and time of seroreversion are unknown. The seroreversion correction model described in the next section uses the estimate of $p^{+}\left[n\right]$ to calculate the seroprevalence accounting for seroreversion.

The criteria to select repeat blood donors were: (1) at least one positive test, indicating SARS-CoV-2 infection, (2) at least one subsequent blood sample, in order to interpolate the date of seroreversion, and (3) falling S/C between these two samples, because one of the samples used to define the interpolation curve may have occurred before the peak S/C; hence, the half-life and the date of seroreversion cannot be estimated. Therefore, all selected donors had at least one positive sample and at least one subsequent sample (positive or negative) with smaller S/C.

To calculate $p^{+}\left[n\right]$, we first estimate the date of seroreversion for each repeat blood donor using an exponential interpolation (a linear interpolation in the log scale). We choose an exponential interpolation because an exponential decay is frequently used to model antibody dynamics (Takahashi et al., 2021). When seroreversion is interval-censored, i.e., a donor that has a positive test subsequently becomes negative, we interpolate an exponential curve that passes through the last positive sample and the first negative sample. Otherwise, when seroreversion is not interval-censored, then it is right-censored (a donor remains positive on their last sample), in which case we extrapolate an exponential line through the last two positive samples and project this forward. As such, the estimated instant of seroreversion for blood donor i (denoted as ${t_{i}}^{-}$) is the point where the interpolation curve crosses the threshold for a positive test. The interpolation procedure is illustrated in Appendix 1—figure 10. The proposed method may overestimate ${t_{i}}^{-}$ if an S/C used to define the interpolation curve was sampled shortly after seroconversion before the peak S/C was reached, since in this case the S/C curve does not behave as an exponential, leading to an overestimated half-life. To partially overcome this problem, we discard donors that do not serorevert within 106 weeks (2 years) after their first positive test.

After estimating ${t_{i}}^{-}$, for each blood donor i, we compute the probability distribution of the date of seroconversion for that donor, $p_{i}$. For this, we identify the earliest and latest possible date of seroconversion $t_{\text{min}}$ (the date of the last negative result before seroconversion or 1 March 2020 if the donor has no positive results before seroconversion) and $t_{\text{max}}$ (the date of the first positive result). The relative probability of seroconversion within this window depends on the incidence of seroconversions due to SARS-CoV-2 infection for the cohort of repeat donors, denoted $u_{\text{repeat}}\left[n\right]$. To estimate this quantity, we calculate the histogram of the date of first positive donation for repeat blood donors and then apply a 30-day moving average. As a sensitivity analysis, we also calculate $u_{\text{repeat}}\left[n\right]$ by computing the histogram of the date of onset of ion (SARI) deaths observed in Manaus, and applying to it a 7-day window moving average, yielding similar seroprevalence estimates (Appendix 1—figures 11 and 12).

The distribution of the date of seroconversion is obtained by truncating the incidence curve of repeat blood donors $u_{\text{repeat}}\left[n\right]$ in the interval $\left[t_{\text{min}},t_{\text{max}}\right]$ and renormalizing the distribution. We then generate 1,000 samples of the instant of seroconversion $t_{i}^{+}∼p_{i}[n]$ and compute the 1,000 sample delays between seroconversion and seroreversion $\Delta t_{i}={t_{i}}^{-}-{t_{i}}^{+}$.

The probability of the delay between seroconversion and seroreversion being n≥1 days (denoted as $p_{\text{day}}^{-}\left[n\right]$) is calculated with the empirical histogram of the $1000\times N_{\text{donors}}$ samples of $\Delta t_{i}$ , $i=1,\cdots ,N_{donors}$. The distribution $p_{\text{day}}^{-}\left[n\right]$ is then binned into weeks by taking the average of $\left(p_{\text{day}}^{-}\left[7n\right],\;p_{\text{day}}^{-}\left[7n+1\right],\cdots ,p_{\text{day}}^{-}\left[7n+6\right]\right)$ for n≥1. The resulting distribution, denoted as $p_{\text{week}}^{-}\left[n\right]$, represents the probability of seroreversion exactly n weeks after seroconversion.

Finally, the probability of a donor remaining positive n weeks after seroconversion $p^{+}\left[n\right]$ (i.e. the probability of a donor seroreverting after week n) is obtained through,

$$p^{+}\left[n\right]=1-\sum_{k=1}^{n}p_{\text{week}}^{-}\left[k\right].$$

The presented method is summarised in Appendix 1.

### Estimating the seroreversion probability from convalescent plasma donors

Unlike repeat blood donors, convalescent plasma donors have a known date of symptom onset. To compute $p^{+}[n]$ for plasma donors, we estimate the instant of seroreversion for each plasma donor as described above and define the date of seroconversion as 8 days after the reported date of symptom onset. This interval of 8 days is the average lag between seroconversion and seroreversion reported in Orner et al., 2021 for a threshold of 1.4 S/C, but it can be shorter for a threshold of 0.49 employed in this work. The probability mass function of the time to seroreversion $p_{\text{day}}^{-}\left[n\right]$ is then the empirical histogram of $\Delta t_{i}={t_{i}}^{-}-{t_{i}}^{+}$, and $p^{+}\left[n\right]$ is obtained from $p_{\text{day}}^{-}\left[n\right]$ using the method presented above.

### Our proposed seroreversion correction model

Here, we present a Bayesian model that draws posterior samples from the incidence over time corrected by sensitivity, specificity, and seroreversion using as input the estimated curve for $p^{+}[n]$, the number of weekly positive tests, and total number of tests. Even though the main output of the model is the incidence at week n for age-sex group a (denoted as u[n,a]), the seroprevalence at week n for group a can be calculated from u[n,a] as $\rho \left[n,a\right]=\sum_{k=1}^{n}u[k,a]$. For simplicity, the proposed model ignores the delay between infection and seroconversion, as it should have small impact on the estimate of u[n,a]. To define the age-sex groups, age was discretized in the intervals 16–24, 25–34, 35–44, 45–54, and 55–69.

Assuming that the sensitivity $se_{0}$ and specificity sp of the assay are independent of the age-sex group, the probability of a random person from age-sex group a being tested positive at week n, denoted as θ[n,a], is

$$\theta \left[n,a\right]=se_{0}\sum_{k=1}^{n}p^{+}[n-k]u[k,a]+\left(1-sp\right)\left(1-\sum_{k=1}^{n}u[k,a]\right).$$

The derivation of the expression above is presented in Appendix 1. The left term $se_{0}\sum_{k=1}^{n}p^{+}[n-k]u[k,a]$ represents true positives (previously infected donors that are still seropositive), while the right term $\left(1-sp\right)\left(1-\sum_{k=1}^{n}u[k,a]\right)$ represents false positives (uninfected donors that test positive).

Let us denote as $T^{+}[n,a]$ and $T\left[n,a\right]$, respectively, the number of positive tests and the total number of tests for week n and age-sex group a. Given θ[n,a], the probability distribution of $T^{+}[n,a]$ is

$$T^{+}\left[n,a\right]|\theta \left[n,a\right]∼Binomial\left(T\left[n,a\right],\theta \left[n,a\right]\right).$$

We use a Bayesian framework to draw posterior samples from u assuming a non-informative prior, but limiting the final seroprevalence in the interval [0,b], where b is a fixed input of the algorithm that can be 1 or 2 depending on whether reinfections are allowed, and we use b=2 in this work. Instead of defining a prior distribution for u[n,a] directly, we decompose it into $u\left[n,a\right]=\rho_{\text{max}}\left[a\right]u_{\text{norm}}\left[n,a\right],$ where $\rho_{\text{max}}\left[a\right]∼Uniform\left(0,b\right)$ sets the upper bound of the final prevalence to b and $u_{\text{norm}}\left[:,a\right]∼Dirichlet\left(1,1,\ldots ,1\right)$ is the normalised incidence which sums to 1. This decomposition is equivalent to assuming a uniformly distributed prior for $u\left[:,a\right]$ in the simplex $0\leq \sum_{n=1}^{N}u\left[n,a\right]\leq b$ with $u\left[n,a\right]\geq 0\forall n$.

After drawing posterior samples from u[n,a], we calculate the seroprevalence at week n for age-sex group a as $\rho \left[n,a\right]=\sum_{k=1}^{n}u[k,a]$ and then compute the age-sex weighted seroprevalence ρ[n], given by

$$\rho [n]=\sum_{a=1}^{M}w\left[a\right]\rho \left[n,a\right],w[a]=\frac{pop[a]}{\sum_{k=1}^{M}pop[k]},$$

where pop[a] is the population for the age-sex group a in the corresponding city and M is the number of age-sex groups. Of note, in this work we also refer to ρ[n] as the estimated seroprevalence, cumulative seroprevalence or attack rate.

The presented Bayesian model is summarised in Appendix 1. The posterior samples are drawn using a Monte-Carlo Markov Chain algorithm with 100,000 iterations.

The incidence returned by the model was validated through posterior predictive checks by randomly selecting 1,000 samples from $u\left[n,a\right]$ and drawing samples from $T^{+}\left[n,a\right]|\theta \left[n,a\right]∼Binomial\left(T\left[n,a\right],\theta \left[n,a\right]\right)$ . The resulting crude seroprevalence is then compared with the measured crude seroprevalence (Appendix 1—figure 13).

It is worth noting that the age-specific crude seroprevalence can be larger than the seroprevalence corrected for seroreversion in some weeks, as the model may remove outlier samples. This is because seroprevalence curves that cannot be reconstructed by the model (e.g. due to bias or sampling noise) generate a small likelihood, hence, a smaller probability of being included in the set of posterior samples generated by the model. Therefore, the model excludes weeks where donors are significantly biased towards more seropositive or more seronegative individuals.

The proposed Bayesian seroreversion correction model can be seen as an improvement on that presented in Buss et al., 2021. The model in Buss et al. assumed a parametric form for time to seroreversion and derived the parameters by assuming an increasing cumulative seroprevalence in the repeated cross-sectional samples of blood donors in Manaus. Here, we derived the distribution directly from repeat blood donors without assuming any parametric form. Also, Buss et al. applied the seroreversion correction method to the measured seroprevalence corrected for sensitivity, specificity, and reweighted by age and sex, while here we estimate the seroprevalence in each age group separately, allowing the identification of non-homogeneous incidence in different age groups.

Despite these differences, the results presented here are compatible with the seroprevalence estimates of 28.8 and 76.0%, respectively, for São Paulo and Manaus in Buss et al. The proposed seroreversion method also differs from other methods in the literature (Shioda et al., 2021; Takahashi et al., 2021) in that we use the incidence curve to estimate the time-dependent sensitivity instead of the deaths or confirmed cases curve, producing a seroprevalence that does not depend on case reporting and that can be reliably inferred in epidemics where the IFR changes with time, as was the case in Manaus.

### Estimating the IFR for December 2020

We estimate the IFR using total deaths due to Severe Acute Respiratory Infection (SARI), which includes PCR- and clinically confirmed SARS-CoV-2 infection as well as SARI deaths without a final diagnosis, and we exclude SARI deaths confirmedly caused by other aetiologies. This approach reduces under-reporting, particularly in 2020 when testing was not widely available, as discussed in de Souza et al., 2020. We further justify this approach in Appendix 1.

We retrieved the daily number of SARI deaths from SIVEP-Gripe (Sistema de Informação da Vigilância Epidemiológica da Gripe), a public database containing individual-level information of all SARI cases reported in Brazil. To estimate the IFR in 2020, we use the seroprevalence estimated by our model for 16 December 2020 and select only SARI deaths with symptom onset between 1 March and 15 December 2020. Selecting deaths based on the date of first symptoms instead of date of death was possible because SIVEP-Gripe contains the date of symptom onset for each individual. For the first wave of COVID-19 that occurred in the eight cities, we estimate the number of cases as the age-specific population size (https://demografiaufrn.net/laboratorios/lepp/) multiplied by the estimated seroprevalence in the corresponding age group. We propagate the uncertainty in the prevalence estimate through the calculation of IFR.

Let ρ[a] and $pop\left[a\right]$ be the cumulative seroprevalence and the population estimated for age group a. We assume a uniform distribution in the interval [0, 1] as a non-informative prior for IFR[a], and the number of deaths D[a] observed for each age group a is Binomial-distributed with size $\left\lfloor \rho \left[a\right]\times pop[a]\right\rfloor$ (the number of infections) and probability IFR[a]. For each sample of ρ[a], we draw a sample of the posterior distribution of $IFR\left[a\right]$ , given by

$$Beta(1+D\left[a\right],1+\lfloor \rho \left[a\right]\times pop\left[a\right]\rfloor -D[a])$$

and compute the median, interquartile ranges (IQRs), and 95% confidence intervals of the IFR by retrieving the quantiles of the posterior distribution.

To infer the IFR, we considered the age groups 16–24, 25–34, 35–44, 45–54, and 55–64. We applied the same method to estimate the overall IFR but using a single age group containing all individuals aged between 16 and 64. Therefore, IFR of individuals older than 64 or younger than 16 is not included in the overall IFR estimates. The method used to infer the IFR was also applied to compute the infection hospitalisation rate (IHR), but we used the number of hospitalisations with SARI instead of the number of deaths.

We note that the proposed seroreversion correction model can be used to estimate the attack rate and IFR of epidemics driven by other lineages in other regions. However, the uncertainty of the seroprevalence estimate increases over time, as a larger amount of seroreversion needs to be corrected. Therefore, estimated attack rates and IFRs suffer from larger uncertainty when longer time periods are considered.

To validate the obtained IFRs, we also estimate the IFRs using the measured prevalence corrected only by the sensitivity and specificity of the assay, without explicitly accounting for seroreversion. In this validation analysis, we use a small threshold of 0.1 S/C to avoid underestimating the prevalence due to seroreversion (see Appendix 1).

### Estimating the IFR for the Gamma VOC

We estimate the IFR and the attack rate separately for the second, Gamma-dominant, SARS-CoV-2 wave that occurred in Manaus. The Gamma variant was first detected in Manaus in November 2020, and its prevalence among PCR-positive patients grew rapidly to 87.0% on 4 January 2021 (Faria et al., 2021). For this reason, it is reasonable to assume that all infections in Manaus that occurred after 15 December, 2020, are due to the Gamma VOC. The Gamma-dominated wave was characterised by a non-negligible proportion of reinfections (Coutinho et al., 2021; Faria et al., 2021; Prete et al., 2022). It is estimated that 13.6–39.3% of the infections in the second wave of COVID-19 epidemic in Manaus were reinfections (Prete et al., 2022), which are explained by the higher in-vitro reinfection potential of Gamma (Lucas et al., 2021) and partial immunity waning 8 months after the first surge. Thus, to calculate the attack rate and IFR of the Gamma-dominated wave, reinfections must be considered.

However, estimating the incidence of reinfections among positive donors is not straightforward - as a positive result may be either primary infection or reinfection, and these cannot be distinguished using a single test result. For this reason, it was not possible to obtain a point estimate for the number of infections that happened in the second wave in Manaus. To overcome this problem, we calculate upper bounds for the attack rate of the Gamma-dominated wave in Manaus (i.e. the incidence between December 2020 and March 2021) and conversely lower bounds for the IFR of the Gamma VOC.

We first estimate the attack rate of the second wave using a Bayesian model that does not take reinfections into account. This model also neglects seroreversion for individuals infected during the second wave due to the small interval of 3 months considered in this analysis (see Appendix 1 for a complete description of the model). Denoting as $\hat{AR}$ the attack rate estimated by this model, the true attack rate AR is given by $AR=\hat{AR}+R+S$, where R is the proportion of donors that were seropositive in December 2020 and subsequently had a reinfection, and S is the proportion of donors that were seropositive in December 2020 and became seronegative in the following months. Since R+S cannot be greater than the seroprevalence in December 2020 (denoted as $\rho_{December}$), the upper bound for the attack rate is $AR_{max}=\hat{AR}+\rho_{December}$. Therefore, the upper bound is obtained assuming that all individuals that were seropositive in December were later reinfected or were seronegative in March 2021.

To estimate $AR_{max}$, we compute the monthly number of positive tests $T^{+}\left[n\right]$ from December 2020 to March 2021 for each age-sex group, as well as the number of true positives (TP) and false negatives (FN) from convalescent plasma donors and the number of false positives (FP) and true negatives (TN) from the pre-pandemic blood donors cohort in Manaus (Supplementary file 1). The Bayesian model generates posterior samples of the crude monthly incidence and the crude seroprevalence in December $\rho_{December}$ . We then correct the crude incidence by the sensitivity of the assay to obtain posterior samples of $\hat{AR}$, which are then added to the posterior samples of $\rho_{December}$, resulting in samples of $AR_{max}$. As explained above, the lower bound for the IFR is then calculated using the upper bound of the attack rate and the number of deaths with symptom onset between 16 December and 15 March. This procedure is repeated for each age-sex group independently and is summarised in Appendix 1.

Only small estimates of the upper bound for the attack rate are informative, as in scenarios where $\rho_{December}$ is small. To limit $\rho_{December}$, we estimate the incidence using a threshold of 1.4 S/C (the upper threshold recommended by the manufacturer) instead of 0.49 S/C (the lower threshold recommended by the manufacturer) and correct for sensitivity based on 163 true positives and 30 false negatives in the plasma donors cohort. Since the specificity of the test using a threshold of 1.4 is 99.9%, and since it is not straightforward to take the specificity into account when reinfections are allowed, we do not correct for specificity in this analysis.

We additionally computed the IFR obtained using the seroprevalence estimated by the model. It is worth noting that our seroreversion correction model only estimates the incidence among seronegative individuals, thus an S/C boosting due to reinfection is not detected by our method. As such, our model estimates the seroprevalence assuming there are no reinfections among positive individuals, underestimating the size of the second wave in Manaus.

The IHR for the Gamma VOC was estimated using the same procedure but using the number of hospitalisations by SARI instead the number of deaths.

This method can be applied to estimate upper bounds for the attack rate of epidemics driven by other lineages with high rates of reinfection such as Delta and Omicron VOCs, but as previously highlighted the upper bound is only informative if the initial crude seroprevalence is small. This may not be the case in regions where vaccines inducing anti-N antibodies were applied, as it is not possible to distinguish vaccination from natural infection based only on anti-N serological data.

### Definition of the homestay index

The homestay index for the eight cities was extracted from https://bigdata-covid19.icict.fiocruz.br/. It was calculated using data from Google Mobility reports using the procedure described in Barreto et al., 2021. The homestay index is defined as

$$Homestay\;Index=X_{H}-\frac{X_{G}+X_{P}+X_{T}+X_{R}+X_{W}}{5},$$

where $X_{H},X_{G},X_{P},X_{T},X_{R}$, and $X_{W}$ are, respectively, the variation of mobility (using pre-pandemic mobility levels as baseline) in the following place categories: residential areas, grocery and pharmacy, parks, transit stations, retail and recreation, and workplaces.

### Calculation of age-standardised estimates

In this work, we calculated the age-standardised mortality, the age-standardised overall IFR, and the age-standardised overall IHR. The procedure used to perform age standardisation was the same for all these quantities. We define an age-standardised variable as the estimate that would be obtained if all cities had the same age structure. Denoting η[a] as an age-specific IFR or IHR for a given city and w[a] as the proportion of the combined population of all eight cities belonging to age group a, then the age-standardised overall IFR or IHR is $\sum_{a=1}^{M}w[a]\eta [a],$ where M=5 is the number of age groups. Similarly, denoting μ(t,a) as the mortality for age group a and day t for a given city, the age-standardised mortality is $\sum_{a=1}^{M}w\left[a\right]\mu (t,a).$

## Results

### Serology assay validation and antibody waning

Antibody kinetics vary with disease severity (Buss et al., 2021; Lumley et al., 2021; Takahashi et al., 2021), and whole blood donors represent predominantly asymptomatic or mild SARS-CoV-2 infections due to donation eligibility criteria (Buss et al., 2021). As such, we sought to estimate a time-to-seroreversion distribution that accurately reflected the blood donor convenience sample used in this study. We identified and tested 7675 repeat whole blood donors in Manaus who had made multiple donations throughout for 2020–2021 (Appendix 1—figure 14) and used these data to estimate the time-to-seroreversion probability distribution (see Materials and methods).

**Figure 1.**
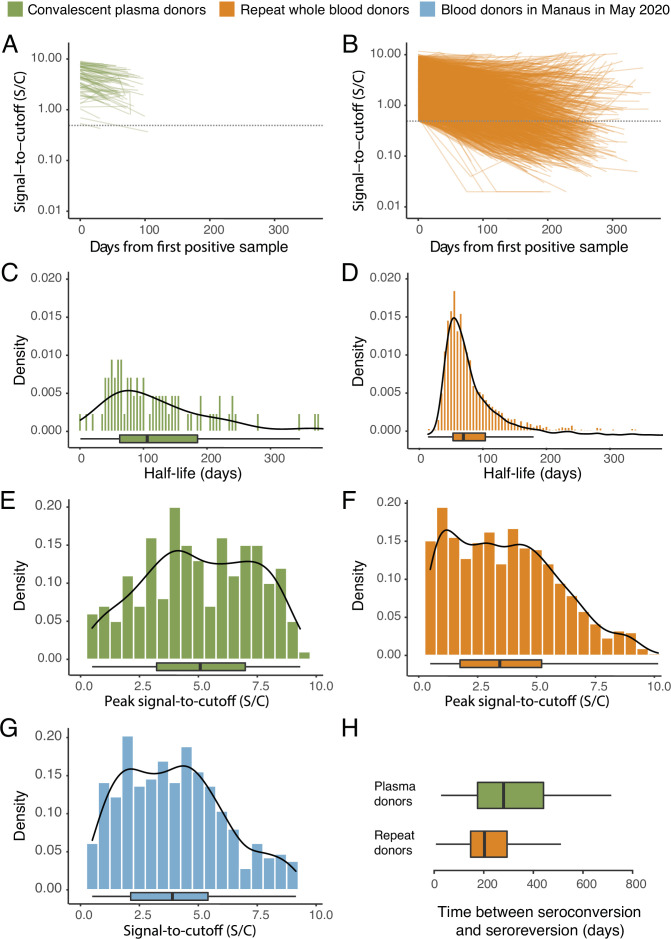
SARS-CoV-2 anti-nucleocapsid (N) IgG dynamics in mild and moderate disease cohorts. (**A**) and (**B**) Trajectories of signal-to-cutoff (S/C) values for the Abbott anti-N chemiluminescent microparticle immunoassays in 218 SARS-CoV-2-infected convalescent plasma donors (**A**) and 7675 repeat whole blood donors (**B**). Time is measured from the first positive test. (**C**) and (**D**) Probability distribution of the half-lives following infection in SARS-CoV-2-infected convalescent plasma donors (**C**) and repeat whole blood donors (**D**). Binned (bars) and smoothed kernel (lines) densities are shown. (**E**), (**F**), and (**G**) Comparison of the probability distribution of the highest S/C measured in plasma donors and seropositive repeat blood donors that donated before 31 May 2020 and the S/C distribution in Manaus in May 2020. (**H**) Estimated time between seroconversion and seroreversion (positive-negative conversion) at a threshold of 0.49 S/C for repeat blood donors and convalescent plasma donors. In all figures, box plots show the median (central lines), interquartile range (hinges), and range extending to 1.5 times the interquartile range from each hinge (whiskers).

The results are shown in Figure 1, which compares the half-life, peak S/C values, and time-to-seroreversion of repeat whole blood donors to the cohort of symptomatic convalescent plasma donors used to determine sensitivity. Repeat blood donors had a shorter assay signal half-life than plasma donors (median [IQR] 69.3 [53.0–103.8] versus 105.9 [62.7–185.1] days) and a lower observed peak S/C ratio (median [IQR] 2.89 [1.49–4.83] versus 5.08 [3.22–6.99]), yielding a shorter median time between seroconversion and seroreversion (203 [147–294] days versus 280 [175–441] days). This highlights the importance of choosing a time-to-seroreversion distribution that is appropriate for the use case - the rate of waning seen in PCR-confirmed symptomatic disease would have resulted in underestimation of SARS-CoV-2 attack rates.

### COVID-19 mortality across Brazilian capitals

**Figure 2.**
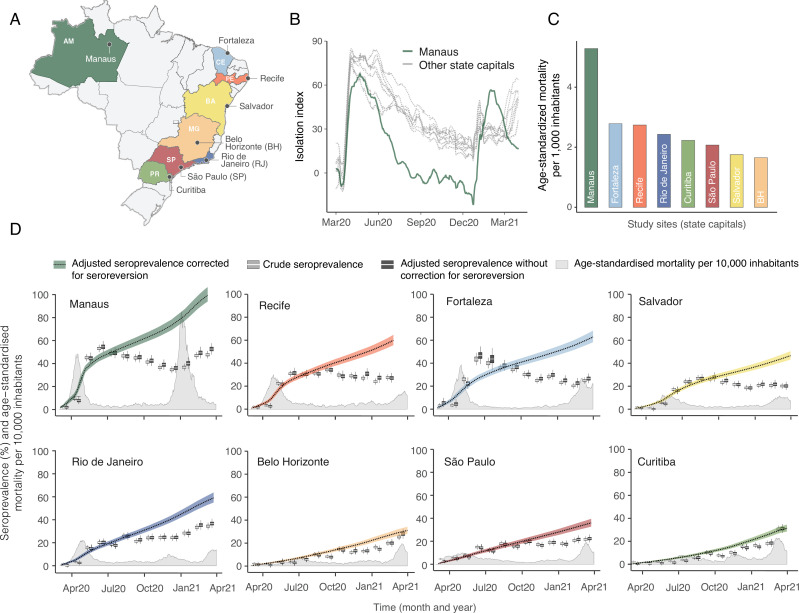
Overview of study site locations, mortality, and mobility data. (**A**) Map of the Brazilian states with the location of the eight capital cities. (**B**) Homestay index for the eight cities. Data were obtained from Fiocruz, available at https://bigdata-covid19.icict.fiocruz.br/. (**C**) Cumulative mortality due to severe acute respiratory syndrome (SIVEP-Gripe system) standardised for age and sex by the direct method using the total Brazilian age-sex structure as reference. Cumulative over the period from 1 March 2020 to 31 March 2021. (**D**) Weekly SARS-CoV-2 seroprevalence in blood donors across eight Brazilian state capitals. Three seroprevalence estimates are shown: (**i**) crude seroprevalence (i.e. the proportion of positive tests); (ii) seroprevalence adjusted for sensitivity, specificity, and reweighted by age and sex but not corrected for seroreversion; and (iii) adjusted seroprevalence estimated by our seroreversion corrected model (continuous curves), which accounts for seroreversion in addition to sensitivity, specificity, and age-sex distribution. Both infections and reinfections in seronegative donors are considered to estimate the adjusted seroprevalence, which can surpass 100%. The grey-filled curve shows age-standardised mortality per 10,000 residents. Ribbons and whiskers represent 95% Bayesian credible intervals.

The location of the eight Brazilian state capitals that contributed serology data is shown in Figure 2A. They collectively represent approximately 14% of the total Brazilian population. The age distributions of the eight cities differ widely (Figure 2—figure supplement 1), as such COVID-19 mortality is presented as age-standardised rates (see Appendix 1—figure 15 for the crude mortality curves). Between 1 March 2020 and 31 March 2021, the age-standardised mortality rate varied from 1.7 deaths per 1,000 inhabitants in Belo Horizonte to 5.3 deaths per 1,000 in Manaus, which had twice the mortality of Fortaleza, the city with the next highest mortality (Figure 2C).

**Figure 2—figure supplement 1.**
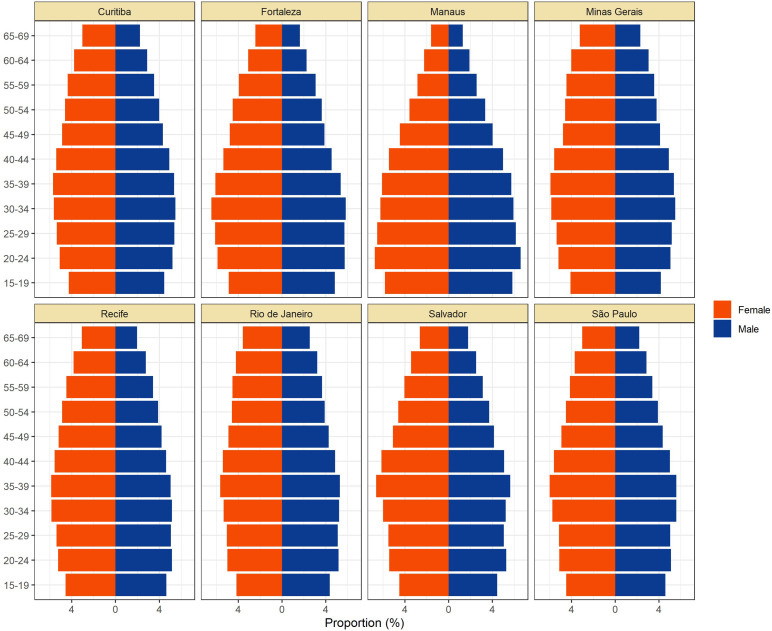
Population pyramids for the eight cities obtained from the projected population estimates for 2020. Data was extracted from https://demografiaufrn.net/laboratorios/lepp/. The proportions are relative to the population between 15 and 69.

**Figure 2—figure supplement 2.**
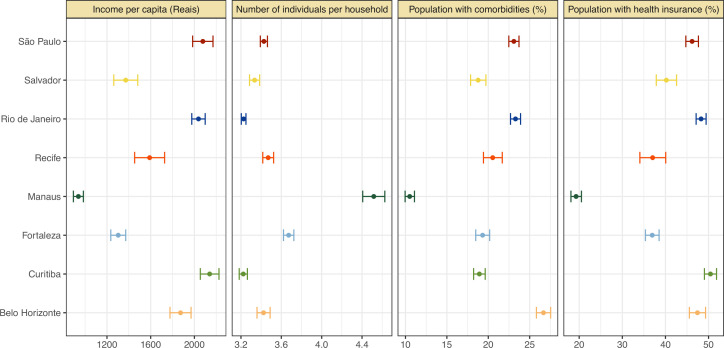
Proportion of population with health insurance, any comorbidity, number of individuals per household, and income per capita for each municipality. The information on health insurance, incidence of comorbidities, number of individuals per household, as well as household income per capita was retrieved from the National Household Sample Survey (Pesquisa Nacional por Amostra de Domicílios COVID-19), a national telephone survey conducted by the Brazilian Institute of Geography and Statistics (IBGE) with over 1,888,560 interviews between May and September 2020. The data on comorbidities included chronic obstructive pulmonary disease, diabetes, hypertension, or cardiovascular disease such as myocardial infarction, angina, or heart failure.

Figure 2B shows the homestay index for the eight cities (see Materials and methods for the definition). Manaus, the city with the youngest population, returned to pre-pandemic levels of mobility by July 2020, having consistently lower homestay index (i.e. higher mobility) than other cities after June 2020, whereas the other seven cities showed a relatively homogenous mobility pattern. The shape of the mortality curves also varied markedly (Figure 2D). Manaus was also an outlier in having the lowest income per capita, health insurance coverage, and lowest proportion of the population with comorbidities, along with the highest number of residents per household (Figure 2—figure supplement 2).

### Blood donor serosurveillance

Using an average of 951 monthly samples of routine whole blood donations (from March 2020 to March 2021, a total of 97,950 samples) in each of the eight cities, we measured the crude seroprevalence of anti-N IgG antibodies detectable by the Abbott CIMA (Table 1). However, these raw estimates of seroprevalence are affected by seroreversion dynamics and provide a poor guide for assessing past levels of population exposure.

**Table 1.** Attack rate estimates for 16 December 2020 (before the Gamma-dominated wave in Manaus) and 24 February 2021. This table contains the attack rate estimated by our seroreversion correction model along with the crude seroprevalence and the adjusted seroprevalence obtained by correcting the crude seroprevalence by sensitivity, specificity, and reweighted by age and sex, but without any correction for seroreversion. Seroprevalence estimates can surpass 100% due to reinfections. Seroprevalence estimates are only available for all cities simultaneously until 24 February 2021.

	December 2020			February 2021		
	Crude seroprevalence (%)	Adjusted seroprevalence with no correction for seroreversion (%)	Attack rate (%)	Crude seroprevalence (%)	Adjusted seroprevalence with no correction for seroreversion (%)	Attack rate (%)
Belo Horizonte	12.8 (10.8–15.1)	13.1 (10.0–16.5)	20.6 (18.6–22.7)	25.2 (22.5–28.1)	27.8 (23.7–32.3)	27.8 (25.2–30.6)
Curitiba	15.2 (13.0–17.5)	14.4 (11.5–17.7)	19.3 (17.5–21.2)	30.8 (28.0–33.7)	31.1 (27.4–35.1)	27.6 (25.2–30.3)
Fortaleza	28.1 (25.4–31.0)	29.8 (25.6–34.2)	48.8 (45.4–52.7)	24.9 (22.2–27.7)	26.6 (22.6–31.0)	57.4 (53.3–62.2)
Manaus	34.6 (31.6–37.7)	36.1 (32.0–40.6)	75.0 (70.8–80.3)	47.7 (44.4–51.0)	52.6 (47.8–57.8)	95.8 (90.6–102.5)
Recife	27 (24.3–29.8)	30.7 (25.9–35.8)	49.4 (46.1–53.1)	27.4 (24.7–30.2)	27.1 (23.2–31.5)	59.9 (55.6–64.6)
Rio de Janeiro	24.4 (21.7–27.2)	24.8 (21.4–28.4)	42.2 (39.4–45.4)	34.5 (31.6–37.5)	36.8 (32.9–41.0)	54.7 (51.1–58.9)
Salvador	18.4 (16.0–21.0)	18.4 (15.3–22.0)	35.3 (32.8–38.1)	20.1 (17.6–22.7)	20.1 (16.8–23.8)	42.4 (39.5–45.8)
São Paulo	18.8 (16.5–21.4)	19.0 (15.9–22.4)	26.6 (24.3–29.1)	21.7 (19.1–24.4)	22.3 (19.0–25.9)	33.3 (30.3–36.5)

Using our Bayesian seroreversion correction model, we present in Figure 2D the age-standardised SARS-CoV-2 attack rates (i.e. the cumulative rate of the population that was infected or reinfected) as of March 2021 after accounting for test sensitivity, test specificity, and IgG seroreversion (coloured lines) along with the directly measured seroprevalence (light grey boxplots) and the estimated seroprevalence adjusting for test sensitivity and specificity (dark grey boxplots). Our results further underscore the significantly different scales of SARS-CoV-2 epidemic impact experienced across the eight cities, with the implied attack rates ranging from only 19.3% in Curitiba, to as high as 75.0% in Manaus by December 2020 (see Table 1). Alternative cumulative seroprevalence estimates produced using different time-to-seroreversion distributions are similar to those in Figure 2D and shown in Appendix 1—figure 12. We note that even though the seroprevalence estimated by our model includes reinfection in seronegative individuals, the model does not capture reinfection in already positive individuals. Therefore, the model is likely to underestimate SARS-CoV-2 attack rates in scenarios where reinfection is not rare, and the obtained seroprevalence can surpass 100% due to reinfections among seronegative individuals.

The slope of the seroprevalence curves (Figure 2D) also differed significantly across cities, showing different dynamics of antibody acquisition at the population level according to the shape and dynamics of the epidemic experienced. Cities with only minimal epidemic peak as Belo Horizonte and Curitiba showed near constant rates of increase in seropositivity after adjustment for antibody waning. By contrast, cities with substantial epidemic peak as Fortaleza and Manaus demonstrated significant variation in the rate at which estimated seropositivity increased in the population, with these rates highest during the epidemic peaks. These findings highlight the capacity of blood donor-based serological data to recapitulate important temporal trends in the intensity and dynamics of the epidemics across these eight cities.

The estimated seroprevalence in June and July in Fortaleza was significantly smaller than the measured seroprevalence without correction for seroreversion, even though the seroprevalence estimates disaggregated by age and sex (Appendix 1—figure 16) lie within or above the confidence intervals of the measured seroprevalence. This effect happened especially in women, which had a crude seroprevalence that was significantly larger than in men in June and July 2020, but became similar in the following months. It is possible that the seroreversion rate observed in Fortaleza had been faster than the rate estimated from repeat blood donors, in which case we undercorrected for seroreversion, underestimating the attack rate. However, a more likely explanation is that samples between March and July 2020 for Fortaleza are less representative of the population, since only 39.4% from 4970 selected samples could have been retrieved and tested, compared to 97.0% for the other cities and months. As such, seropositive individuals from Fortaleza may have been more likely to donate in these months, leading to an overestimated crude seroprevalence.

### Age-sex patterns in blood donor seroprevalence

**Figure 3.**
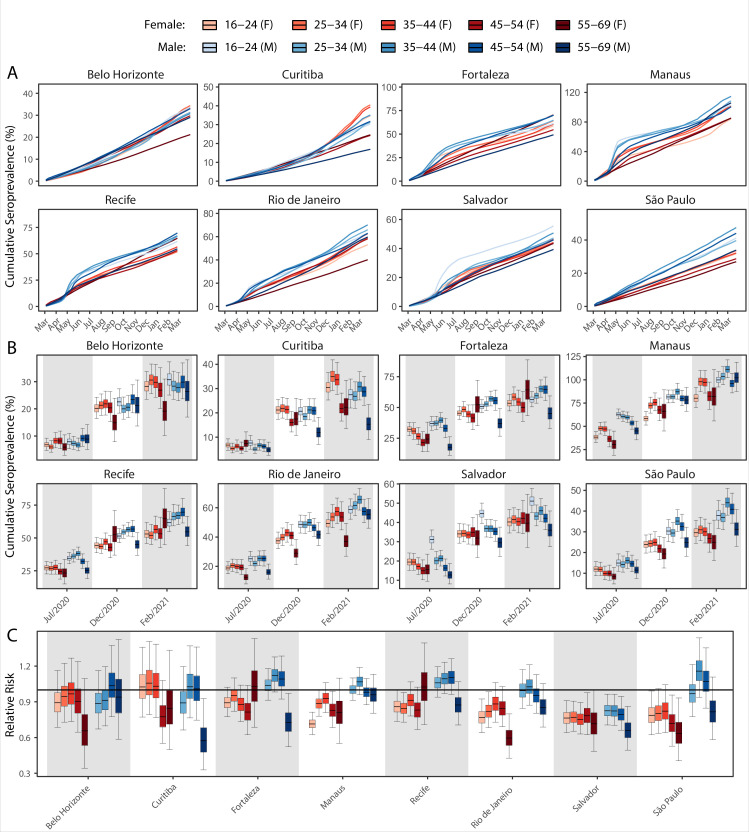
Age-sex patterns in blood donor seroprevalence in eight Brazilian cities. (**A**) Estimated cumulative seroprevalence by age-sex group. (**B**) Transversal cuts of figure (**A**) on 8 July 2020, 16 December 2020, and 24 February 2021 (last week where seroprevalence was estimated for all cities). (**C**) Relative risk of the cumulative seroprevalence estimated in December 2020 with men aged 16–24 as the reference category in each city. Note that since cities use different values as reference, only relative risks of age-sex groups from the same city can be compared. Box plots show posterior distributions of the relative risks, with the median (central lines), interquartile range (hinges), and 95% confidence intervals (whiskers).

We next examine the patterns and dynamics of attack rates across different groups by disaggregating the seroprevalence data by age and sex. The seroprevalence estimates disaggregated by age and sex are shown in Figure 3 (see Appendix 1—figures 17–18 for seroprevalence disaggregated by only age or sex). Across the eight cities, our results consistently show differences between sexes - on average, men tended to have higher attack rates than women, although the degree and extent of this difference varied between cities. In São Paulo, the seroprevalence in December 2020 for men was 30.6% compared to the 23.0% estimated in women (i.e. 33.5% (95% CrI 17.7–51.9) higher, Figure 3B). By contrast, seroprevalence in Curitiba in December 2020 was similar for women and men, being only 4.65% (95% CrI –11.5 to 18.5) higher in women.

We also observed an extensive variation in the dynamics of population-level seroprevalence between age groups, with seroprevalence in December 2020 typically highest in younger age groups. The seroprevalence of individuals below the age of 55 increased in all cities except for Recife when compared to donors aged between 55 and 69, increasing by 34.1% (95% CrI –2.23–91.2) in Curitiba and decreasing by a small factor of 0.5% (95% CrI –24.8–19.1) in Recife. Furthermore, in cities with a large increase in seroprevalence during the first epidemic wave (i.e. Manaus, Recife, Fortaleza, and Salvador), this was primarily driven by younger men. In these locations, the differences between age-sex groups slowly narrowed during the long period of less intense transmission (Figure 3A). This highlights important differences between age-groups in the extent to which they were exposed to the virus and/or contributed to transmission at different points during the regional epidemics - differences that are not evident, or certain, from case or death counts alone.

In addition to the differences in attack rates by age and sex, seroprevalence did not increase homogeneously among different age and sex groups. In Manaus, seroprevalence was significantly larger in men and younger individuals aged 16–44 in July 2020, but between July and December seroprevalence increased faster in women and donors older than 45 years, leading to smaller differences in attack rate by age and sex in December 2020 (Figure 3B). Similar patterns are also observed in Salvador, Recife, and Fortaleza, although with smaller age and sex inequalities.

### Variation in the SARS-CoV-2 IFR across age groups and locations

**Figure 4.**
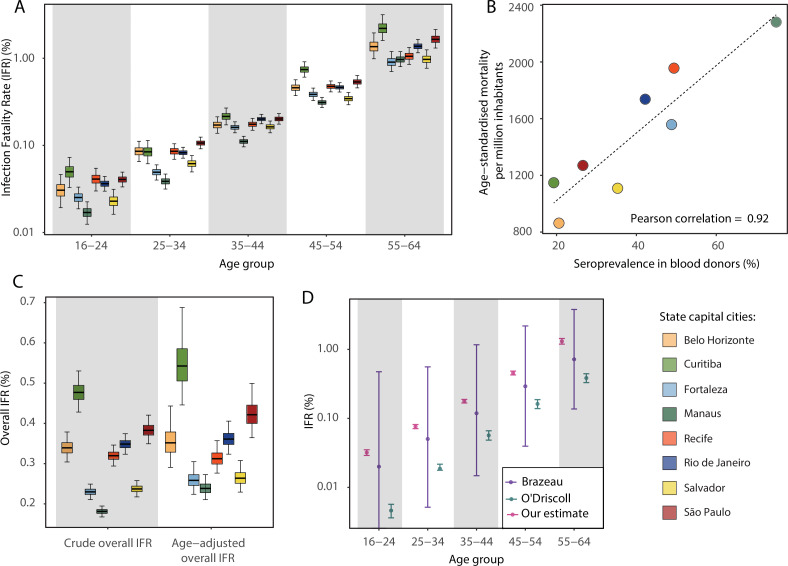
Infection fatality rates (IFRs) in eight Brazilian state capitals as of 15 December 2020. The number of deaths was obtained from the SIVEP-Gripe reporting system including all SARI deaths with symptom onset between 1 March 2020 and 15 December 2021. (**A**) Age-specific IFRs. (**B**) Association between age-standardised mortality rate and cumulative seroprevalence in blood donors for each of the eight cities by December 2020. The black line is a linear regression fit to the coloured points, each representing one of the eight cities. (**C**) Crude and age-adjusted overall IFRs, for the age range of 16–64 years, in each of the eight participating cities. (**D**) Overall IFR of the eight cities for the age range of 16–64 years obtained with our age-specific IFR estimates compared with the overall IFR calculated using age-specific IFRs from Brazeau et al., 2020 and O’Driscoll et al., 2021.

Using estimates of the cumulative number of individuals infected alongside records of COVID-19 deaths available from Brazil’s SIVEP-Gripe platform, we next calculated the IFR for each city and age group. Figure 4A presents the estimated age-specific IFRs for each municipality as of December 2020, before the Gamma VOC epidemic in Brazil. Our results show the IFR significantly increases with age, ranging from 0.03% in individuals aged 16–24 years to 1.31% in individuals aged 55–64 years. This is in-keeping with previous work highlighting a strong age dependency in COVID-19 mortality (Brazeau et al., 2020; Buss et al., 2021; O’Driscoll et al., 2021). Cities presented different age-standardised overall IFRs, being smaller in Manaus (0.24%) and higher in Curitiba (0.54%).

**Figure 4—figure supplement 1.**
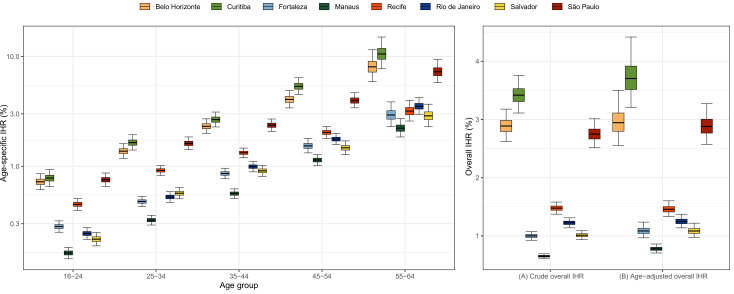
Estimated age-specific and overall infection hospitalisation rates (IHRs) for the eight cities in the period between March and December 2020. Note that IHR depends not only on intrinsic disease severity but also on access to healthcare and availability of hospital resources, which can vary across cities.

There was a strong correlation (Pearson’s correlation = 0.92) between the age-standardised mortality rate in each city and the attack rate inferred from blood donor serosurveillance data (Figure 4B). Both the overall IFR and the overall IFR adjusted for the age structure of the city differed significantly between cities (Figure 4C), showing that the IFR differences cannot be explained only by the different age structures. Despite the differences between cities, the obtained age-specific IFRs were similar to the estimates from Brazeau et al., 2020 but higher than the estimates from O’Driscoll et al., 2021 (Figure 4D). The age-specific and overall IHR were also estimated (Figure 4—figure supplement 1) and showed similar patterns, being larger in Belo Horizonte, Curitiba, and São Paulo.

The obtained IFRs and attack rates for December 2020 were validated using alternative approaches that do not correct directly for seroreversion, not depending on the proposed seroreversion correction model (see Appendix 1).

### The dynamics and epidemiological impacts of the Gamma VOC in Manaus

As previously highlighted, we could not obtain a point estimate of the attack rate in the Gamma-dominated period in Manaus because we are unable to identify which of the seropositive blood donors are primary infections and which are reinfections. Instead, we calculated upper bounds assuming maximum proportions of reinfections. The inferred upper bound of the age-specific attack rate in the Gamma-dominated period in Manaus ranged from 30.6% (95% Bayesian CrI 22.8–41.1) to 46.0% (95% CrI 32.8–60.6) in individuals aged 45–54 and 55–64 (Figure 5—figure supplement 1), showing small variation among age groups. The estimated upper bound for the age-standardised cumulative attack rate in the second period dominated by the Gamma variant was 37.5% (95% CrI 35.3–42.6), significantly smaller than the cumulative attack rate of 75.0% (Figure 2) estimated for the first period dominated by non-Gamma variants.

**Figure 5.**
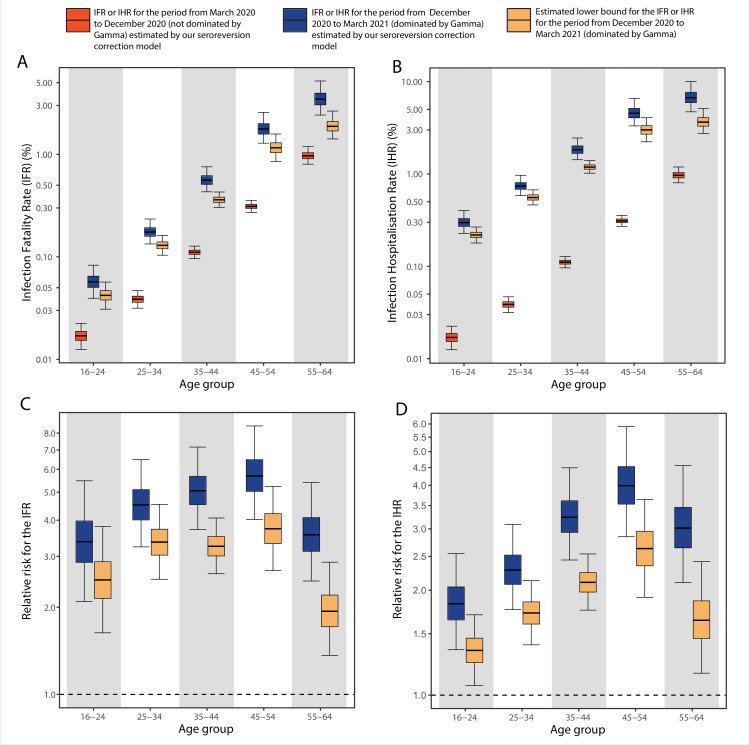
Comparison of infection-to-hospitalisation and infection-to-fatality rate estimates during the non-Gamma and Gamma period in Manaus. (**A**) Estimated infection fatality rates (IFRs) and (**B**) infection hospitalisation rates (IHR s) for Manaus in the periods from 1 March 2020 to 15 December 2020 (non-Gamma dominated) and 16 December 2020 to 31 March 2021 (Gamma dominated). For the Gamma-dominated period, estimates shown are lower bounds that were calculated assuming a maximum proportion of reinfections (see Materials and methods). (**C**) Relative risks of the lower bound estimate of the iIFRs in the Gamma-dominated period using the estimated IFRs in the non-Gamma period as reference and (**D**) similarly for IHRs.

Comparing to the COVID-19 attributable hospitalisations and deaths reported to the SIVEP database, we next used the estimated upper bounds of the age-specific attack rates in the Gamma period in Manaus to calculate lower bounds of the age-specific IHR and IFR for the Gamma period. We then compared the IFRs and IHRs obtained with the attack rate estimated for the period during which non-Gamma variants dominated (from 1 March 2020 to 15 December 2020). The resulting age-specific IFRs and IHRs are shown in Figure 5A, B, respectively, and the relative risks obtained using the IFR or IHR in December 2020 as baseline in Figure 5C, D. The lower bound for the IHR increased in all age groups, from 34.4% (95% CrI 6.5–70.0) in individuals aged 16–24 to 163.4% (95% CrI 90.9–264.3) in individuals aged 45–54 when compared to the IHR estimated for the non-Gamma period. The increased hospitalisation risk combined with an increased in-hospital fatality rate (HFR, defined as the number of deaths per hospitalisation) during the second wave (Appendix 1—figure 19) resulted in an increased age-specific IFR, with a lower bound increasing 93.8% (95% CrI 36.4–186.4) in individuals aged 55–64 to 273.5% (95% CrI 167.8–423.4%) in individuals aged 45–54 when compared to the first wave (Figure 5C). As such, even though the IFR and IHR increased for all age groups during the Gamma-dominated period, this difference was more significant in younger age groups. The obtained lower bound for the overall IFR was 0.527% (95% CrI 0.447–0.630), 2.91 (95% CrI 2.43–3.53) times higher than the estimated IFR for the first wave in Manaus.

**Figure 5—figure supplement 1.**
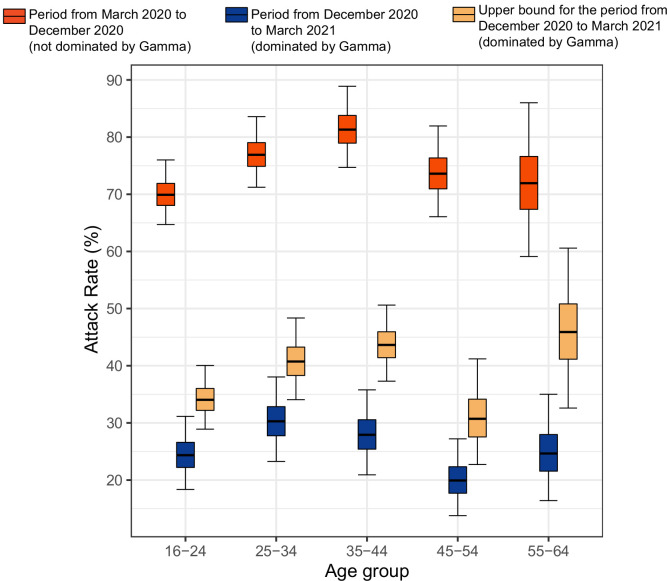
Attack rate in Manaus estimated by our seroreversion correction model for the periods March 2020–December 2020, December 2020–March 2021 and the estimated upper bound for the attack rate.

## Discussion

Our results highlight the divergent epidemic dynamics across eight of Brazil’s biggest cities as reflected by mortality rates, and show that these differences are recapitulated in blood donor-based serial cross-sectional serosurveillance. Despite the large IFR differences observed across cities, seroprevalence was strongly correlated with cumulative age-standardised mortality (Figure 4B). These results reinforce the validity of blood donors as a convenient population for serosurveillance. A previous study (Mina et al., 2020) has highlighted the need for a reliable, cost-effective method of immunological surveillance to provide evidence of past infection and to understand the dynamics of emerging disease. Even though serology is less precise for identifying infections on an individual level, it is an effective tool for monitoring epidemics at a population level. As blood donation programs are an existing component of medical infrastructure globally and in which blood samples are readily available in many locations, this approach can be rapidly implemented and carried out in large populations.

We estimated larger attack rates in individuals aged 16–54 years. This is consistent with previous work examining age patterns of transmission from mobility data in the United States (Monod et al., 2021), but we have measured infection directly rather than making inferences indirectly on the basis of COVID-19 deaths and movement. Possible reasons for the higher attack rate in people aged 16–54 years include, but are not limited to, different risk perception and shielding practises, and disease biology with more frequent asymptomatic infections in younger people, which increase infection risk in this age group due to greater mixing among working age adults. We also found overall higher levels of seroprevalence in men compared to women, and these patterns changed over time. For instance, in Manaus, a very high seroprevalence was reached rapidly among young men by July 2020, after which relatively little increase in overall seroprevalence occurred in men. By contrast, among older women, who reached less than half the attack rate seen in men by June, the seroprevalence continued to increase. This heterogeneity in transmission in a location with high overall antibody prevalence meant that some groups remained relatively susceptible and perpetuated transmission at a lower level (Buss and Sabino, 2021; Lalwani et al., 2021). Other works suggest that socioeconomic condition also contributed to heterogeneity of SARS-CoV-2 spread in Manaus (Lalwani et al., 2021), which is confirmed by the large seroprevalence observed in Black and less-educated donors (Appendix 1—figure 20).

We also confirm a strong age dependency of COVID-19 IFRs (Brazeau et al., 2020; O’Driscoll et al., 2021). Although age-specific IFRs were roughly similar across the cities (Figure 4A) and similar to estimates in the literature (Figure 4D), there were some noticeable differences. For example, the more affluent south and southeastern cities of Belo Horizonte, Curitiba, and São Paulo tended to have higher age-specific IFRs, whereas in the northern and northeastern cities of Manaus, Salvador, and Fortaleza, the age-specific IFRs tended to be lower. This may be due to under-reporting of deaths but might also reflect lower prevalence of comorbidities in the latter populations (Figure 2—figure supplement 2). Cities with larger IFRs also had larger IHRs, suggesting that the differences in IFR reflect the different risks of developing a severe disease. The different lineages circulating in the eight cities may have also contributed to the observed IFR and IHR difference (Appendix 1—figure 1). While most of the cases in the first wave in Amazonas and Ceará were caused by earlier lineages, the lineages B.1.1.28, B.1.1.33, and later P.2 (Zeta) were more prevalent in other states. It is worth noting the IHR also depends on the probability of an individual with severe disease being hospitalised. This probability depends on access to health facilities and availability of healthcare resources, and therefore may vary across cities even if disease severity remains constant.

Our results also clearly demonstrate a higher IHR during the Gamma-dominated observation period compared to the non-Gamma observation period in Manaus for all age groups (Figure 5). This supports observations (Banho et al., 2021) that the Gamma VOC tends to cause more severe disease than the ancestral non-Gamma variants circulating locally, even among young adults in Manaus. The larger increase in IHR for younger adults aged 25–54 years is compatible with the younger profile of hospitalisations of the Gamma-dominated wave in Brazil (de Souza et al., 2021)^,^ observed before vaccination coverage reached significant levels in older age groups. In Manaus, the increased levels of hospitalisation caused parts of the healthcare system to collapse during the second wave causing an increase in HFR as previously described (Brizzi et al., 2021), further increasing the IFR. The higher IFR associated with Gamma VOC infection during the second wave is therefore due to a combination of two factors – increased disease severity resulting in a greater proportion of infections requiring hospital-based care (the IHR, arising primarily from intrinsic viral properties and pathogenicity), and the impacts of this increased healthcare pressure on mortality within-hospitals (the HFR, arising primarily from healthcare pressure).

There are some relevant limitations to our results that need to be pointed. First, blood donors are a convenience sample, and extrapolation to the entire population should be done with caution. Due to eligibility criteria in Brazil, blood donors are limited to those aged 16–69 years, with a strong skew towards younger adults even within this eligibility range (Figure 2—figure supplement 1) in most Brazilian regions. However, our results do suggest that blood donor serosurveillance agrees with other metrics of epidemic size as mortality, both cumulatively (Figure 5B) and through time (Figure 3). Moreover, both sensitivity and seroreversion could be an age-dependent process as a proxy for disease severity, i.e., older individuals are more likely to be symptomatic, seroconvert, and have longer time to seroreversion. Indeed, we see this pattern of longer half-lives and larger peak S/Cs in convalescent plasma donors who had recovered from more severe disease (Appendix 1—figure 21). Therefore, correction of crude seroprevalence for antibody waning could possibly be confounded by demographic differences between the eight cities. However, since individuals that had a severe disease are unlikely to donate blood, seropositive whole blood donors are likely fairly homogenous in having had milder or asymptomatic disease, and as such, the rate of waning may not vary significantly between locations. An additional important point to note is that the longer an epidemic last, the more frequent reinfections become due to the natural waning of immunity in the time period following infection. Our data span over a year of transmission in areas with multiple waves with high SARS-CoV-2 burden and consequently non-negligible reinfection rates, as such it is difficult to reliably infer the attack rates from seroprevalence data towards the end of the time series. For this reason, our model produces upper bounds for cumulative prevalence of >100% in Manaus by early 2021.

Despite these limitations, blood donors represent an accessible population to detect trends of the epidemic that otherwise could only be obtained through expensive population-based studies, which are difficult to establish in Brazil during the course of a rapidly progressing epidemic. Studies to understand the main differences between blood donors and the general population would help the development of better sampling protocols to mitigate bias and should be part of preparedness for future epidemics of infectious diseases.

## Figures and Tables

**Appendix 1—figure 1.**
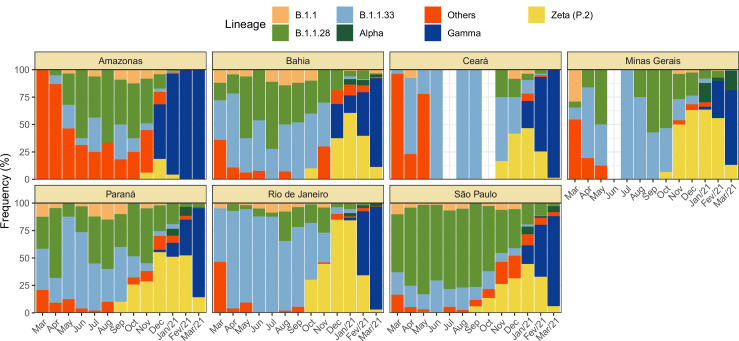
Monthly frequency of each lineage among cases confirmed by PCR for each state from March 2020 to March 2021. Data was extracted from http://www.genomahcov.fiocruz.br/. Lineage data was not available for the state of Pernambuco.

**Appendix 1—figure 2.**
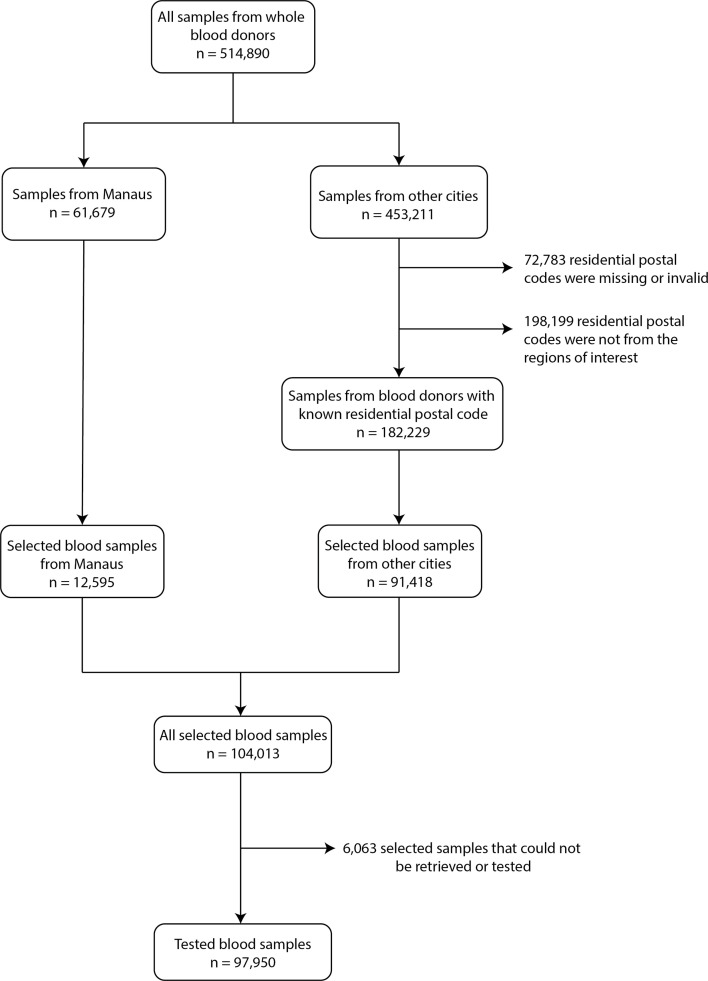
Flowchart describing the procedure used to select blood samples. Samples were selected based on the residential postal code of blood donors to generate a representative set of test results. In Manaus, samples were selected consecutively without any postal code restriction, as postal codes could not be reliably collected in this city.

**Appendix 1—figure 3.**
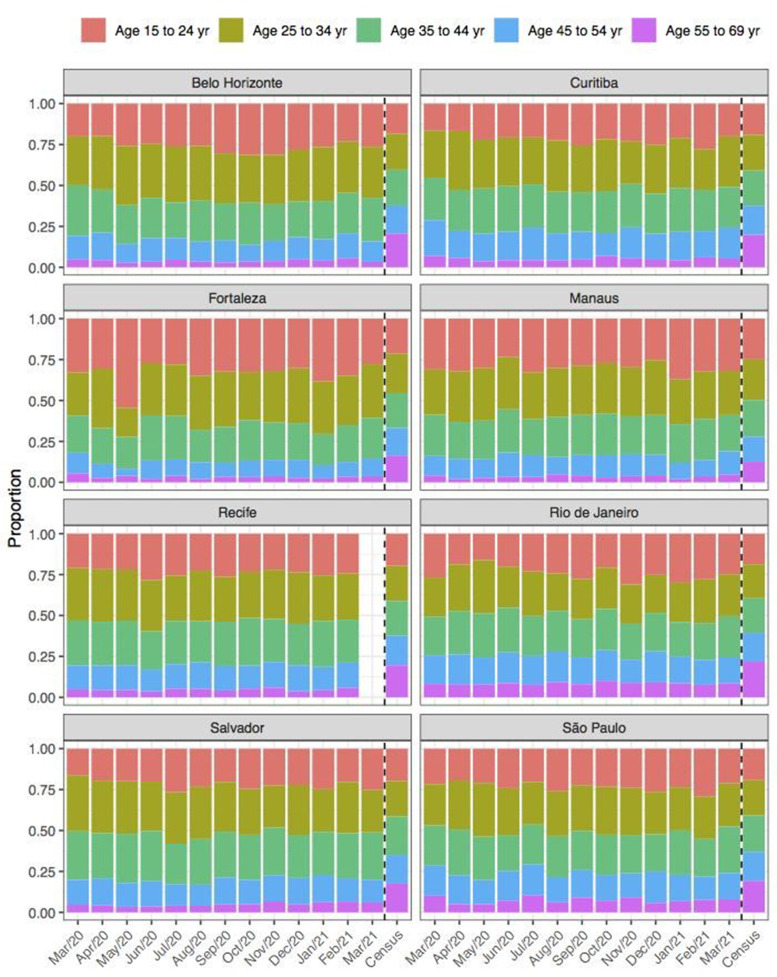
Comparison of age structure in blood donors tested for SARS-CoV-2 IgG antibodies population size projections for 2020 based on the last available 2010 census.

**Appendix 1—figure 4.**
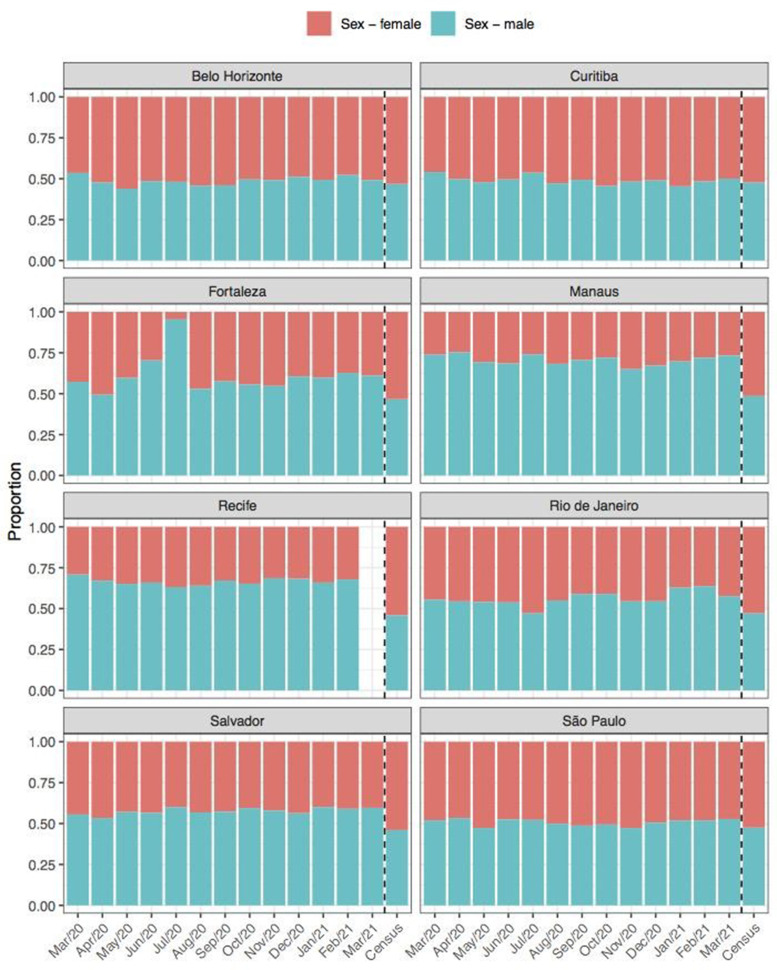
Comparison of sex structure in blood donors tested for SARS-CoV-2 IgG antibodies and the sex distribution at the last Brazilian census (2010).

**Appendix 1—figure 5.**
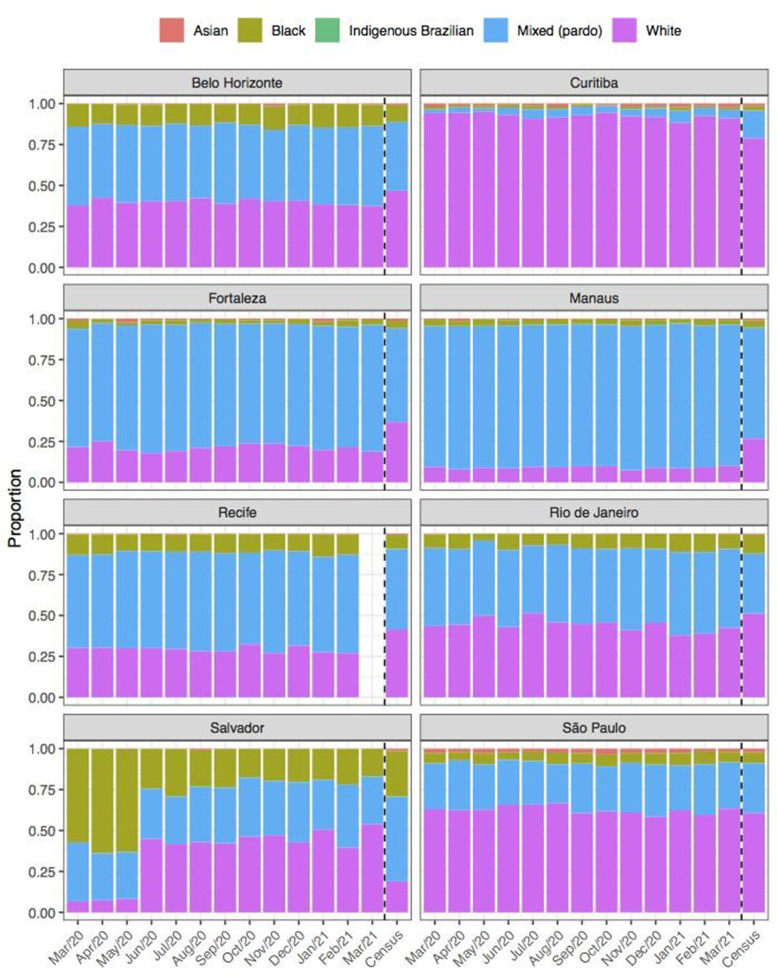
Comparison of self-declared skin colour among blood donors tested for anti-SARS-CoV-2 IgG antibody and the distribution of skin colour at the last available Brazilian census (2010).

**Appendix 1—figure 6.**
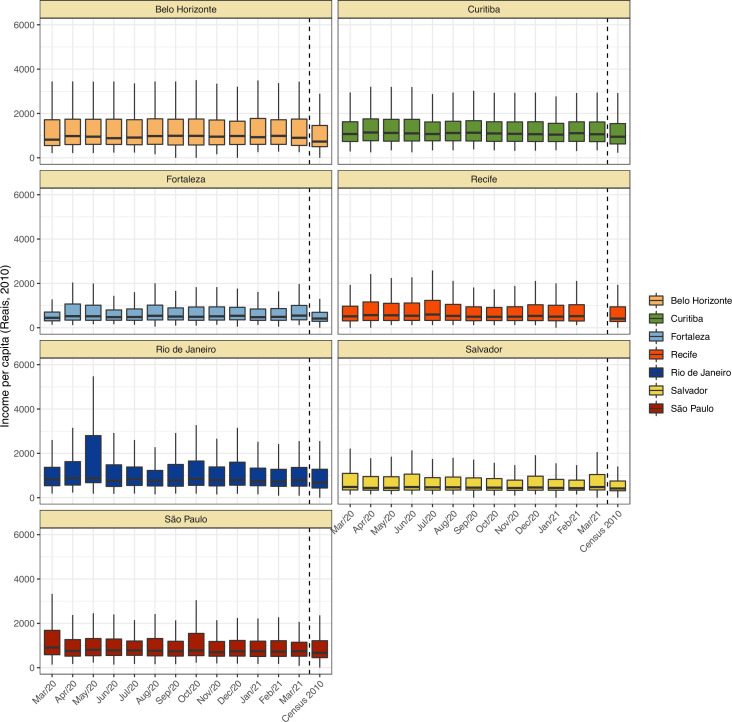
Comparison of income per capita of the census tract of selected blood donors and the income per capita distribution at the last available Brazilian census (2010) for each municipality. Information on census tracts was not available for Manaus.

**Appendix 1—figure 7.**
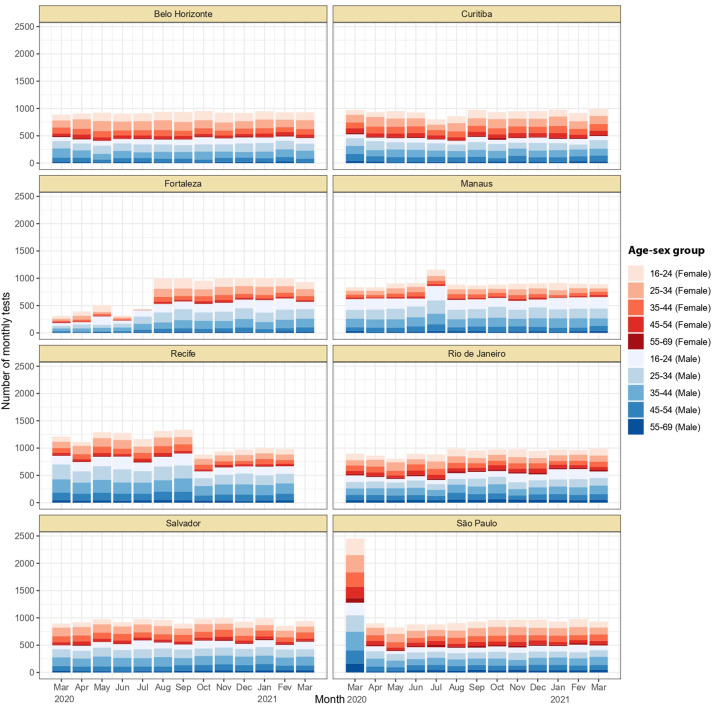
Monthly number of tests disaggregated by age group and sex. Around 1,000 monthly tests were applied between March 2020 and March 2021, except for Recife where tests were applied until February 2021.

**Appendix 1—figure 8.**
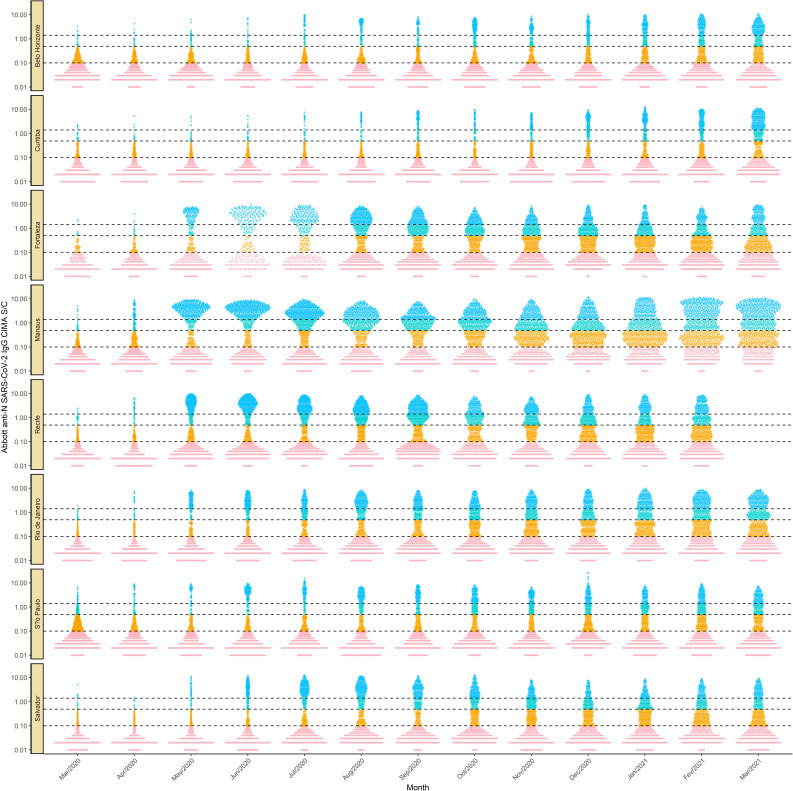
Monthly antibody signal-to-cutoff (S/C) reading in each of the eight cities. Each point represents the test result of a blood donor. The dashed lines represent the thresholds 1.4 (the threshold recommended by the manufacturer), 0.49 (the lower threshold recommended by the manufacturer and used in the main analyses of this paper), and 0.1, which we use in validation analyses.

**Appendix 1—figure 9.**
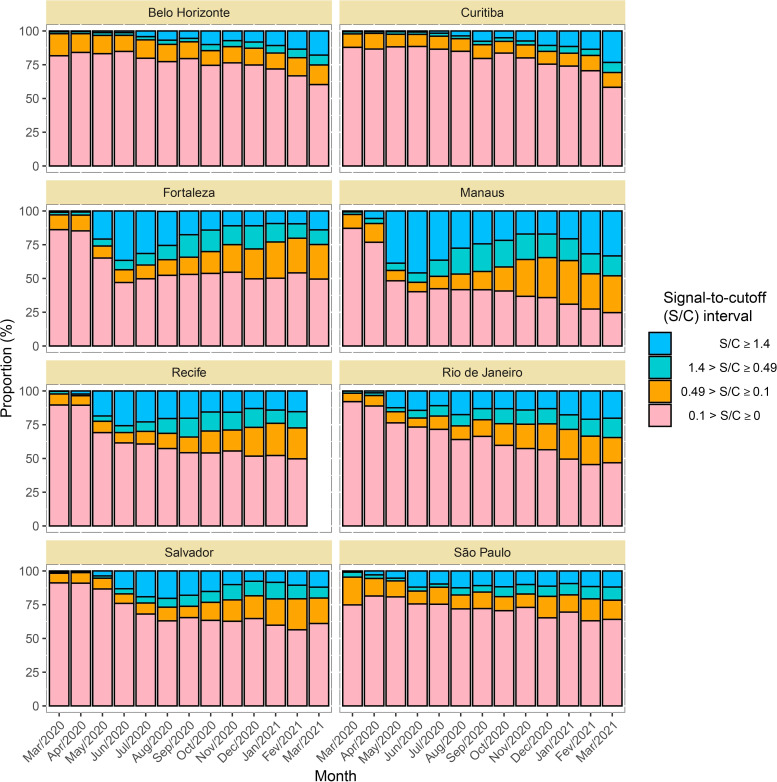
Time series of the proportional distributions of raw signal-to-cutoff (S/C) readings in monthly blood donor samples. The thresholds were chosen as follows: 1.4 is the manufacturer’s recommended upper threshold for assay positivity, which maximises specificity; 0.49 is the manufacturer’s lower recommended threshold, which improves sensitivity following antibody waning; and 0.1 S/C is an even lower threshold used for this analysis, which still provides specificity of 86% (112 false positives in 821 pre-pandemic blood donation samples) but further improves sensitivity in the face of antibody waning.

**Appendix 1—figure 10.**
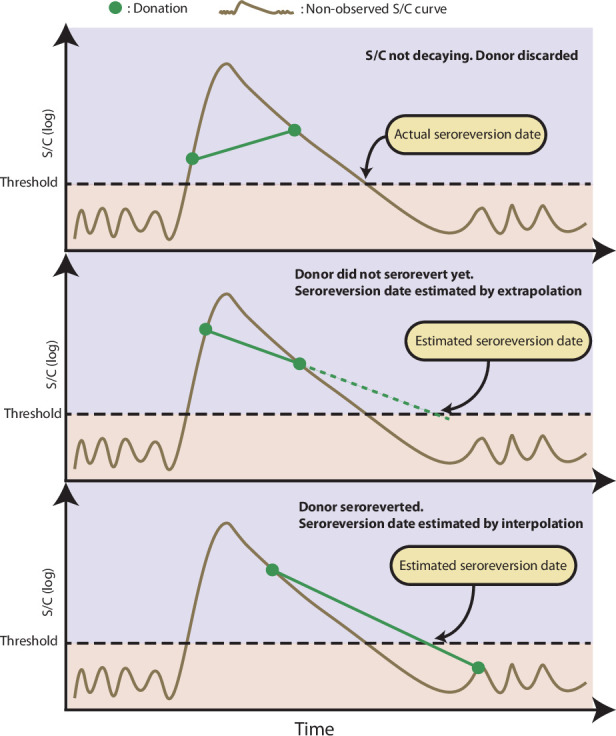
Illustration of the procedure used to estimate the time of seroreversion for each repeat blood donor. This figure shows three donors with the same idealized signal-to-cutoff (S/C) curve but different donation dates. The first donor was discarded because the observed S/C was rising. The second donor did not become negative yet after seroconversion, thus the seroreversion date is estimated by extrapolating an exponential curve that contains the last two positive results. The third donor became negative after seroconversion; hence, the seroreversion date was estimated by applying an exponential interpolation that contains the last positive result and the first negative result after seroconversion.

**Appendix 1—figure 11.**
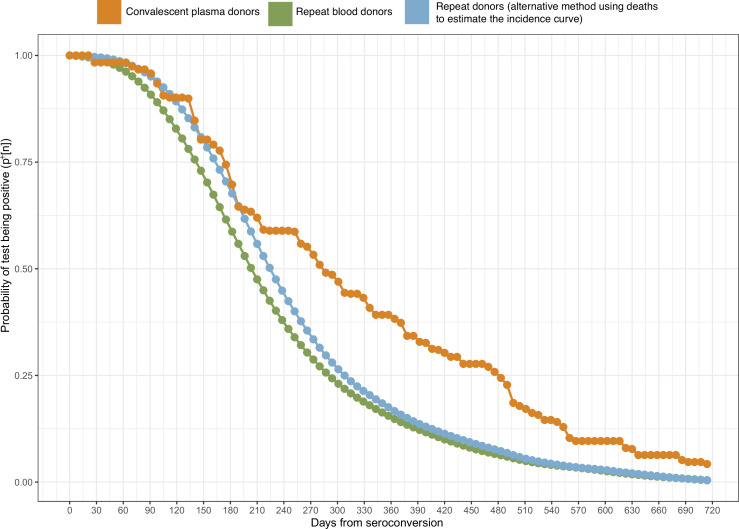
Probability of a test being positive a given number of weeks after seroconversion given that seroconversion occurred $p^{+}[n]$ in terms of the method used to estimate it. In this work, we use the estimate of $p^{+}[n]$ obtained from repeat blood donors (green curve).

**Appendix 1—figure 12.**
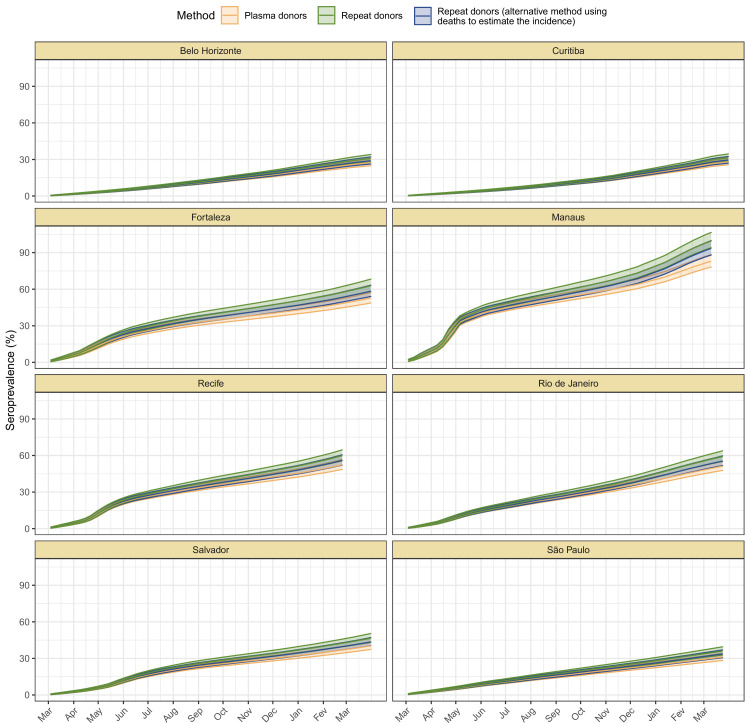
Cumulative seroprevalence estimated with 95% credible intervals (ribbons) using three different methods to calculate the time-to-seroreversion distribution. For the analyses in this paper, we consider the seroprevalence curve in green, obtained using repeat donors to estimate the time to seroreversion.

**Appendix 1—figure 13.**
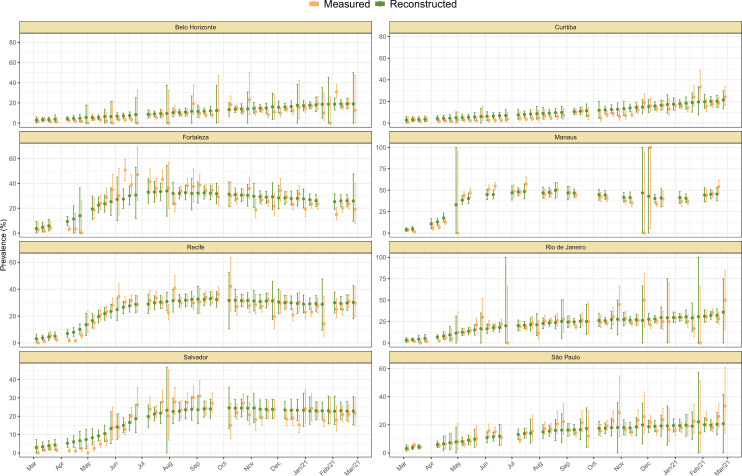
Measured weekly crude seroprevalence compared to the crude seroprevalence reconstructed by our proposed seroreversion correction model. The high similarity between both quantities shows that the seroprevalence estimated by the model is compatible with the observations.

**Appendix 1—figure 14.**
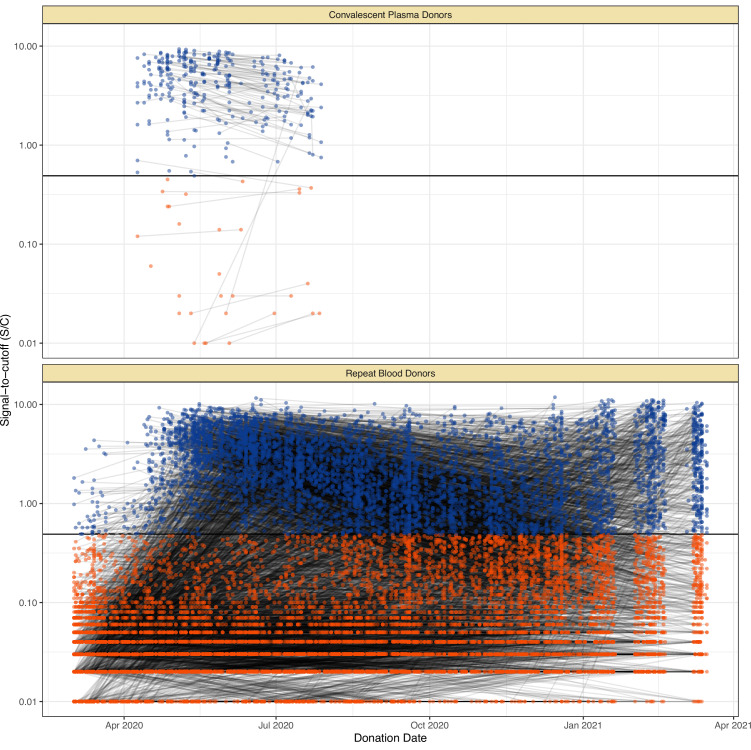
Serial donations of 218 convalescent plasma donors (symptomatic and known date of onset, PCR-positive) and 7675 repeat whole blood donors (unknown if symptomatic or date of onset, unknown PCR status) included in this study. These cohorts were used to determine the rate of antibody waning and time-to-seroreversion distributions for the anti-nucleocapsid chemiluminescent microparticle immunoassays.

**Appendix 1—figure 15.**
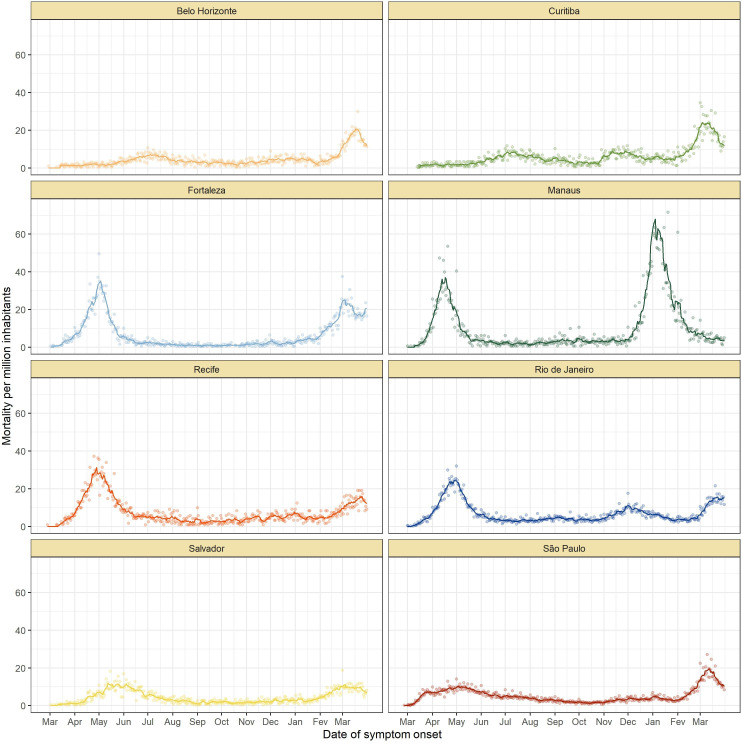
Mortality by severe acute respiratory infection per million inhabitants in each of the eight cities without any age standardisation.

**Appendix 1—figure 16.**
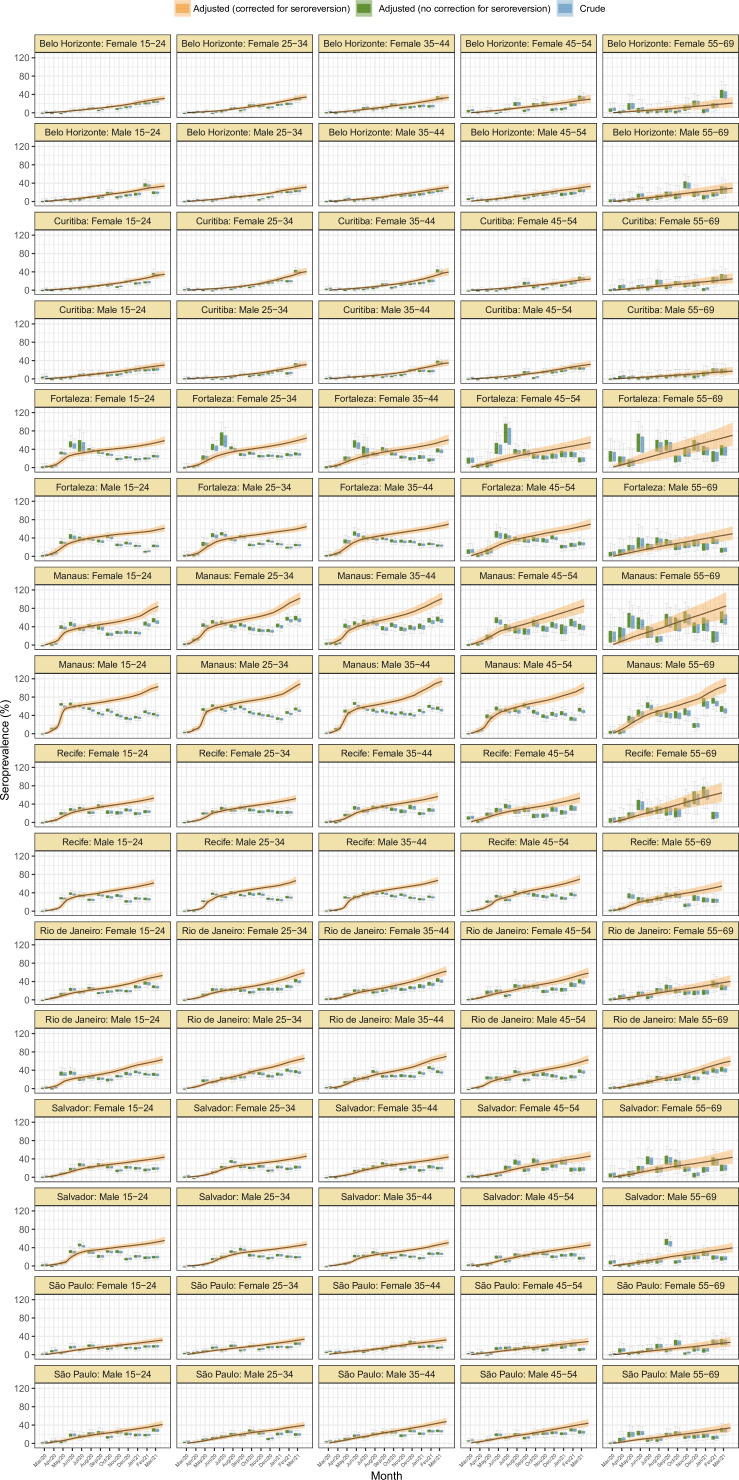
Seroprevalence estimates disaggregated by age and sex for the eight cities. As in Figure 2D, three seroprevalence estimates are shown: (**i**) crude seroprevalence; (ii) seroprevalence adjusted for sensitivity, specificity, and reweighted by age and sex, but not corrected for seroreversion; and (iii) adjusted seroprevalence estimated by our seroreversion corrected model (continuous curves), which accounts for seroreversion in addition to sensitivity, specificity, and age-sex distribution. This seroprevalence estimate consists in the sum of infections and reinfections among seronegative donors; hence, it can be larger than 100%.

**Appendix 1—figure 17.**
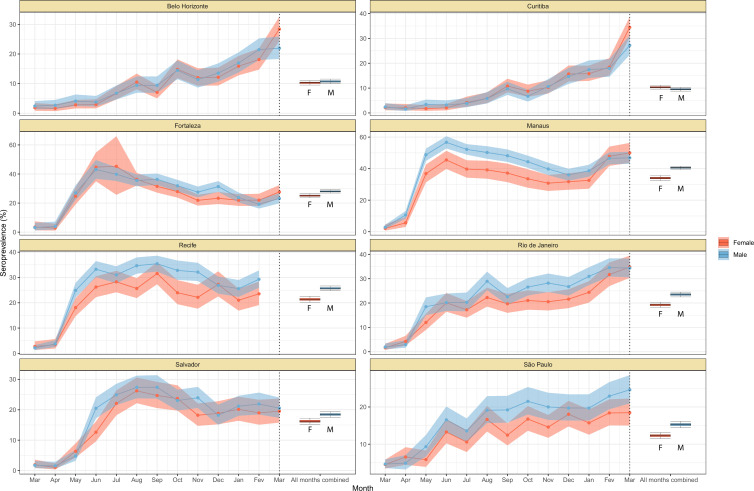
Crude monthly seroprevalence disaggregated by sex. Boxplots represent the crude seroprevalence obtained by aggregating all months, defined as the proportion of positive tests combining all months.

**Appendix 1—figure 18.**
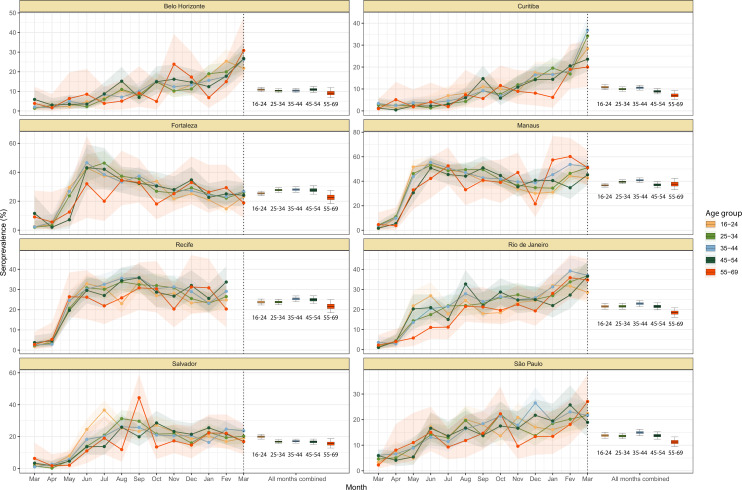
Crude monthly seroprevalence disaggregated by age. Boxplots represent the crude seroprevalence obtained by aggregating all months, defined as the proportion of positive tests combining all months.

**Appendix 1—figure 19.**
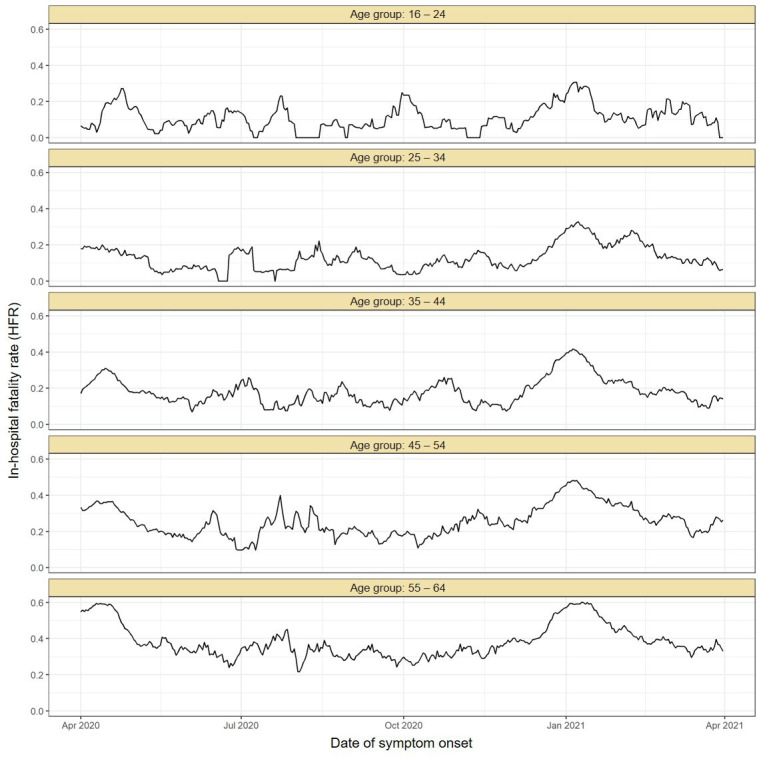
Estimated in-hospital fatality rate (HFR) over time in Manaus. The HFR was calculated by dividing the number of deaths by the number of hospitalisations using a moving window of 14 days. The HFR had a peak in the Gamma-dominated wave in January 2021 in all age groups.

**Appendix 1—figure 20.**
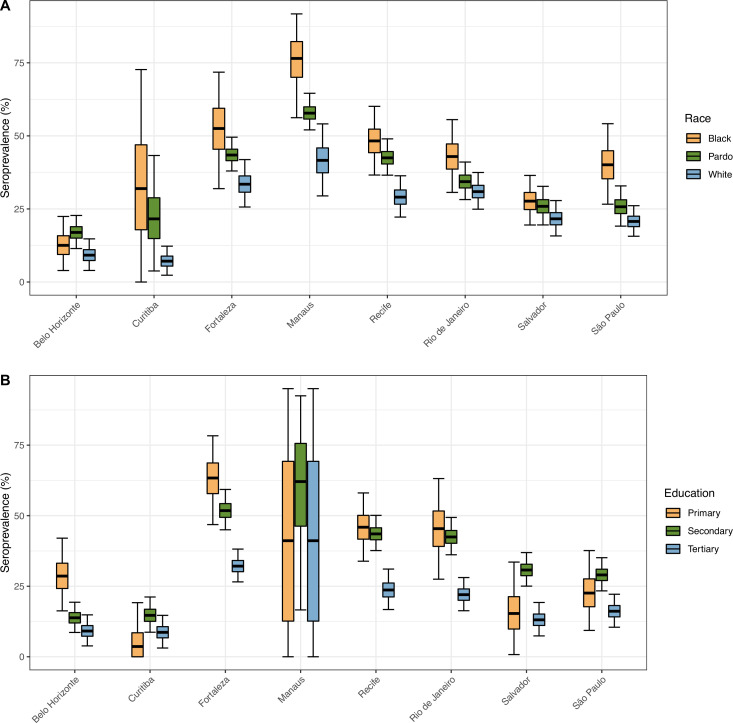
Seroprevalence in December 2020 disaggregated by race and education level. The seroprevalence was estimated using a threshold of 0.1 signal to cutoff and correcting for sensitivity and specificity, without any explicit correction for seroreversion. To aid visualization, we merged the White and East Asian races and discarded Indigeneous individuals. Information on education level is available for very few donors in Manaus, hence, the large confidence intervals.

**Appendix 1—figure 21.**
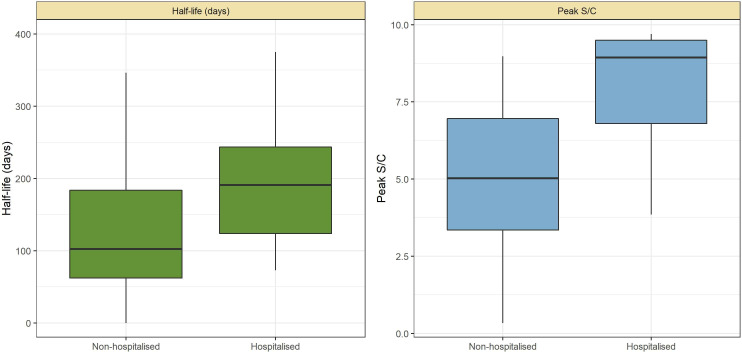
Abbott SARS-CoV-2 anti-nucleocapsid half-life and peak signal to cutoff (S/C) estimated in convalescent plasma by hospitalisation status. Instead of estimating one half-life for each plasma donor, a different half-life was calculated for each pair of consecutive donations with decaying S/C reading. Box plots show the median (central lines), interquartile range (hinges), and range extending to 1.5 times the interquartile range from each hinge (whiskers).

**Appendix 1—figure 22.**
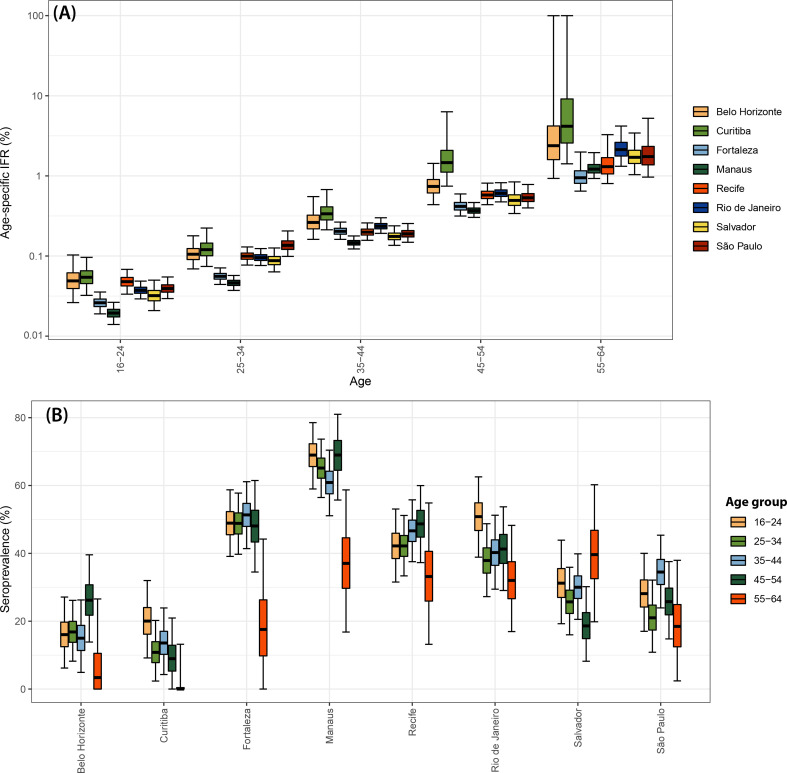
Infection fatality rate (IFR) and seroprevalence measured in December 2020 using 0.1 as threshold and correcting for sensitivity, specificity, and reweighting by age and sex. No seroreversion correction was performed to estimate the seroprevalence. That the wide confidence intervals for the IFR of Curitiba and Belo Horizonte are due to the small number of infections and deaths in these cities.

**Appendix 1—figure 23.**
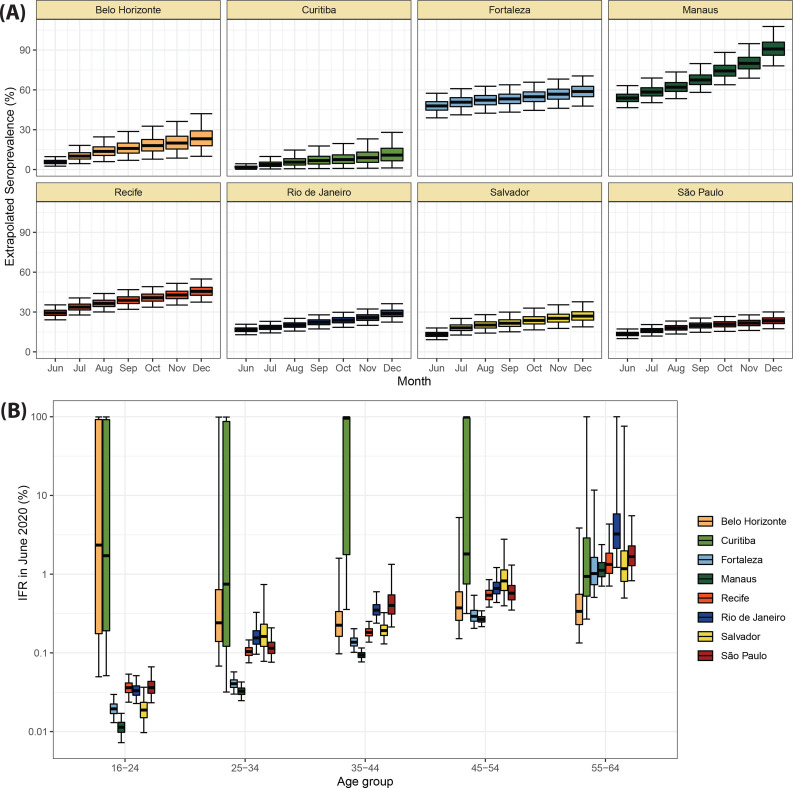
Validation of the seroprevalence estimates using infection fatality rate (IFR) extrapolation. (**A**) Seroprevalence obtained by extrapolating the IFR measured in June 2020 to the following months. The seroprevalence was obtained by dividing the cumulative number of deaths in each month by the IFR estimated for June 2020. (**B**) Age-specific IFR estimated in June 2020. Cities with small attack rate in June 2020 as Belo Horizonte and Curitiba present a large uncertainty in the estimated IFR.

**Appendix 1—figure 24.**
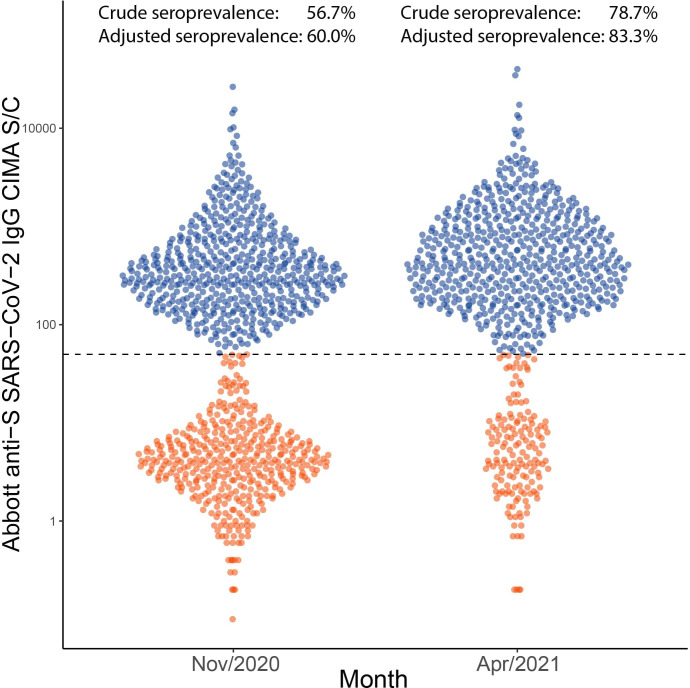
Distribution of signal to cutoff (S/C) in Manaus obtained in November 2020 and April 2021 with the anti-spike assay. Each point represents the test result for a blood donor. Using a threshold of 50 (dashed line), the crude seroprevalence was 56.7% in November and 78.7% in April. Adjusting for sensitivity, these estimates increase to 60.0 and 83.3%.

**Appendix 1—figure 25.**
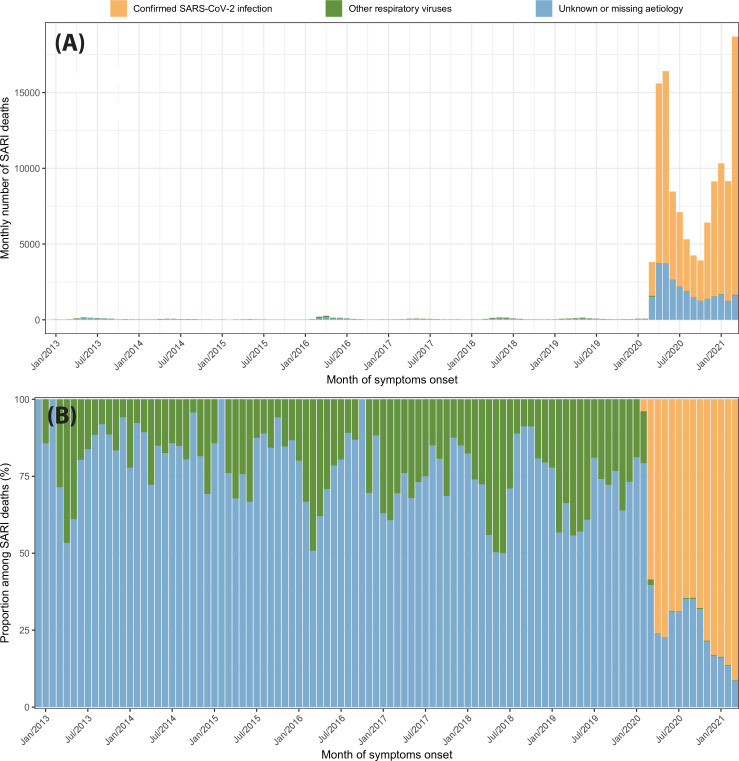
Severe acute respiratory infection (SARI) deaths disaggregated by case classification over time. (**A**) Monthly number of SARI deaths in the eight cities for each case classification recorded in the SIVEP-Gripe dataset from 2013 to 2021. (**B**) Proportion of SARI deaths following each classification among all SARI deaths recorded in that month.

**Appendix 1—figure 26.**
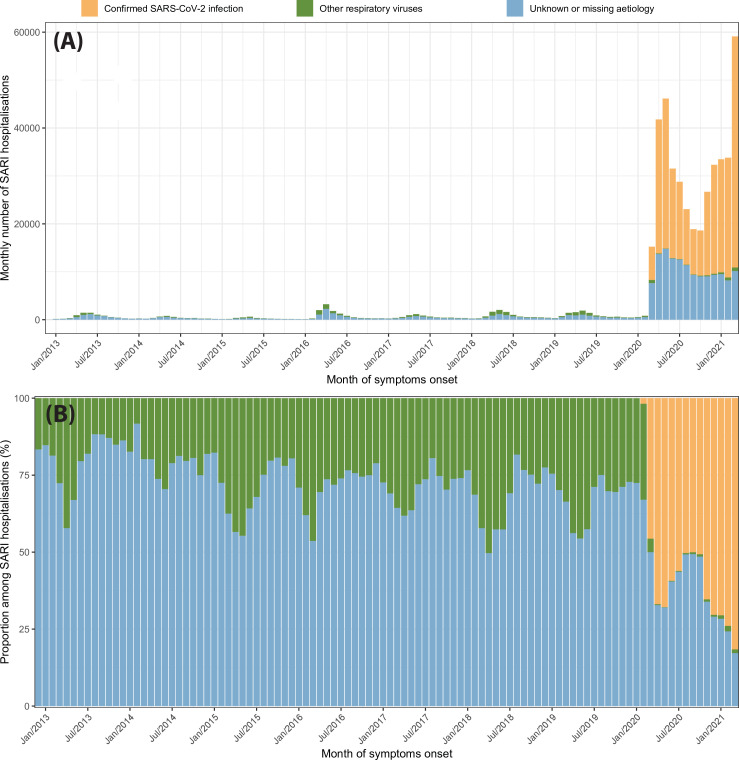
Severe acute respiratory infection (SARI) hospitalisations disaggregated by case classification over time. (**A**) Monthly number of SARI hospitalisations in the eight cities for each case classification recorded in the SIVEP-Gripe dataset from 2013 to 2021. (**B**) Proportion of SARI hospitalisations following each classification among all SARI hospitalisations recorded in that month.

## Data Availability

All serological data required to reproduce the analyses are available at Data Dryad (doi:https://doi.org/10.5061/dryad.dz08kps08) and can be downloaded at https://datadryad.org/stash/dataset/doi:10.5061/dryad.dz08kps08. The codes used for the main analyses are available at https://github.com/CADDE-CENTRE/seroprevalence_eight_cities, (copy archived at swh:1:rev:67518ad26368c1f4856fdfd4c08673abeded4901). The following dataset was generated: PreteCA BussL WhittakerC SalomonT OikawaM PereiraR MouraI DelerinoL Barral-NettoM TavaresN FrançaR BoaventuraV MiyajimaF Mendrone-JuniorA de Almeida NetoC SallesN FerreiraS FladzinskiK de SouzaL SchierL InoueP XabregasL CrispimM FraijiN AraujoF CarlosL PessoaV RibeiroM de SouzaR CavalcanteA ValençaM da SilvaM LopesE Amorim FilhoL MateosSO NunesG da SilvaS Silva-JuniorA BuschM CastroM DyeC RatmannO FariaN NascimentoV SabinoE 2022Data from: SARS-CoV-2 antibody dynamics in blood donors and COVID-19 epidemiology in eight Brazilian state capitalsDryad Digital Repository10.5061/dryad.dz08kps08PMC954555636135358

## Additional files

Supplementary file 1. Number of true positives (TPs), true negatives (TNs), false positives (FPs), and false negatives (FN) used to estimate the sensitivity of the anti-nucleocapsid assay for the thresholds 1.4, 0.49, and 0.1 signal to cutoff (S/C), and for the anti-spike assay for the threshold 50.0. Sensitivity and specificity were calculated by computing the quantiles of Beta (1+TP, 1+FN) and Beta (1+TN, 1+FP), respectively.

Supplementary file 2. Age-specific infection fatality rates (IFRs) and 95% credible intervals obtained with the seroprevalence estimated with our seroreversion correction model and with a threshold of 0.1 signal to cutoff corrected for sensitivity and specificity.

MDAR checklist

## Appendix 1

### Validation of the obtained attack rates and IFRs

The seroprevalence and IFRs obtained in December 2020 estimated with our seroreversion correction model were validated using a smaller threshold of 0.1 and correcting only for sensitivity and specificity, without explicitly correcting for seroreversion (Appendix 1—figure 22, Supplementary file 1). Even though this approach underestimates the seroprevalence (thus overestimates the IFR) because a fraction of previously seropositive donors had already seroreverted by December 2020 (leading to a significant number of false negative test results), the obtained attack rates and IFRs were similar to the estimates of our model. The inferred seroprevalence for Manaus and Curitiba in December 2020 was, respectively, 61.0% (95% CrI 56.5–65.4%) and 13.4% (95% CrI 10.0–17.2%), compatible with the estimates of our seroreversion correction model (Figure 2, Table 1) given that these quantities underestimate the seroprevalence due to waning. The IFR pattern across cities was also similar in this analysis, being higher in Curitiba and smaller in Manaus for almost all age groups.

An alternative approach to estimating the attack rate in December 2020, in the face of waning antibodies and falling assay sensitivity, is to calculate the IFR early in the epidemic, prior to significant waning, and extrapolate the number of future cases from the reported death time series. To further validate the attack rates, we calculated the age-specific IFRs in June 2020 (when less seroreversion is expected) for the age range eligible to donate blood and extrapolated using only the deaths within this age bracket. As such, the seroprevalence obtained for the other months is based solely on the number of deaths and the IFR inferred for June 2020 (Appendix 1—figure 23). This approach led to an estimated seroprevalence of 90.8% (95% CrI 78.1–107.7%) and 10.9% (95% CrI 1.2–28.0%) in Manaus and Curitiba, respectively, in December 2020, which are compatible with our estimates if confidence intervals are considered. Nevertheless, this approach has the limitation of assuming a constant IFR through time and only using a small amount of the total available serologic data.

To validate the inferred cumulative attack rate in November 2020 prior to the Gamma-dominated second wave and also in April 2021, following this second wave, we re-tested 996 samples from November 2020 in Manaus and tested 769 samples from April 2021 using the Abbott anti-S SARS-CoV-2 IgG CIMA (Appendix 1—figure 24), which showed less waning than the Abbott anti-N assay used in this work (Stone et al., 2021). As such, the usage of the anti-S assay reduced the difference between the seroprevalence obtained without explicitly correcting for seroreversion and the true seroprevalence. A sensitivity of 94.0% was obtained by testing convalescent plasma donors with this assay (Supplementary file 1), and the specificity was assumed as 100%. The crude prevalence of anti-S antibodies was 56.7% (95% CrI 53.6–59.8%) in November 2020 and 78.7% (95% CrI 75.6–81.4%) in April 2021. After correcting for sensitivity and reweighting by age and sex, the seroprevalence estimate was 60.0% (95% CrI 58.4–62.2%) in November 2020 and 83.3% (95% CrI 81.1–86.4%) in April 2021, compared to 68.0% (95% CrI 64.2–72.7%) and 99.5% (95% CrI 94.0–106.6%) estimated using our seroreversion correction model for November 2020 and March 2021. Note that the attack rate estimated by our model considers both infections and reinfections among seronegative individuals, hence, the confidence intervals higher than 100%. Of note, we measured the half-life of this assay using the serial repeat blood donors data available in Prete et al., 2022, obtaining a median half-life of 124.5 (interquartile range 74.7–258.0) days. In November, 6 months following the first wave in Manaus, some cases of seroreversion are expected to have occurred; as such, this remains an underestimate of the true cumulative attack rate by this point. Assuming no reinfections before November, the smaller seroprevalence measured with the anti-S assay suggests that 8.0% of previously infected donors seroreverted before November 2020.

### Derivation of the expression of the probability of a positive test

In this section, we derive the expression of the probability of a positive test θ[n,a] in terms of the age-specific weekly incidence u[n,a]. Let us denote the negation of an event E as $\bar{E}$, and the probability of an event E as P{E}. To shorten the next equations, we also denote $T^{+}\left(n,a\right)$ the event of a test applied to an individual from age-sex group a at week n being positive, I(n,a) the event of an individual from age-sex group a being infected at week n such that the incidence at week n and age-sex group a is P{I(n,a)}, $I\left(1:n,a\right)={⋃}_{k=1}^{n}I(n,a)$ the event of an individual from group a having being infected before week n and C(a) the event of an infected individual from group a seroconverting after infection. We consider that the initial sensitivity $se_{0}$ (i.e. the sensitivity right after seroconversion) and specificity sp of the assay do not depend on the age-sex group or on time.

The probability $\theta \left[n,a\right]$ of a test applied to a person from age-sex group a being positive at week n is

$$\theta \left[n,a\right]=P\left\{T^{+}\left(n,a\right)\right\}=P\left\{T^{+}\left(n,a\right),I\left(1:n,a\right)\right\}+P\left\{T^{+}\left(n,a\right),\bar{I}\left(1:n,a\right)\right\}.$$

The first term can be decomposed as

$$P\left\{T^{+}\left(n,a\right),I\left(1:n,a\right)\right\}=\sum_{k=1}^{n}P\left\{T^{+}\left(n,a\right),I\left(1:n,a\right)|I\left(k,a\right)\right\}P\left\{I\left(k,a\right)\right\}=\sum_{k=1}^{n}P\left\{T^{+}\left(n,a\right)|I\left(k,a\right)\right\}P\left\{I\left(k,a\right)\right\}.$$

The term $P\left\{T^{+}\left(n,a\right)|I\left(k,a\right)\right\}$ can be further decomposed into

$$P\left\{T^{+}\left(n,a\right)|I\left(k,a\right)\right\}=P\left\{T^{+}\left(n,a\right)|I\left(k,a\right),C\left(a\right)\right\}P\left\{C\left(a\right)|I\left(k,a\right)\right\}+P\left\{T^{+}\left(n,a\right)|I\left(k,a\right),\bar{C}\left(a\right)\right\}P\left\{\bar{C}\left(a\right)|I\left(k,a\right)\right\}.$$

Assuming that an infected individual that did not seroconvert cannot have a positive test at any instant, we have $P\left\{T^{+}\left(n,a\right)|I\left(k,a\right),\bar{C}\left(a\right)\right\}=0$. We approximate $P\left\{T^{+}\left(n,a\right)|I\left(k,a\right)\right\}$ to $p^{+}[n-k]$ (i.e. the probability of a test being positive n-k weeks after seroconversion), neglecting the delay between infection and seroconversion. Since the mean delay between infection and seroreversion is smaller than 8 days as explained above, and since crude seroprevalence data are discretized using weeks as time unit, this delay has small influence on seroprevalence estimates. Because P{C(a)∣I(k,a)} is the sensitivity $se_{0}$ of the assay and P{I(k,a)} is the incidence u[k,a], we have $P\left\{T^{+}\left(n,a\right),I\left(1:n,a\right)\right\}$ $=se_{0}\sum_{k=1}^{n}p^{+}\left[n-k\right]u\left[k,a\right]$.

The second term of θ[n,a] is

$$P\left\{T^{+}\left(n,a\right),\bar{I}\left(1:n,a\right)\right\}=P\left\{T^{+}\left(n,a\right)|\bar{I}\left(1:n,a\right)\right\}P\left\{\bar{I}\left(1:n,a\right)\right\}=\left(1-sp\right)\left(1-\sum_{k=1}^{n}u\left[k,a\right]\right),$$

where $\text{sp}=P\left\{T^{+}\left(n,a\right)|\bar{I}\left(1:n,a\right)\right\}$ is the specificity of the assay, which does not change over time.

Therefore, a simpler expression for θ[n,a] is obtained:

$$\theta \left[n,a\right]=se_{0}\sum_{k=1}^{n}p^{+}[n-k]u[k,a]+\left(1-sp\right)\left(1-\sum_{k=1}^{n}u[k,a]\right).$$

### Description of the method used to validate the seroprevalence and IFR for 2020

To validate the seroprevalence and IFRs estimated for 2020, we recalculate these quantities by measuring the prevalence in December 2020 with a smaller threshold equal to 0.1 to partially account for seroreversion and correct for sensitivity and specificity, without explicitly incorporating a method to correct for seroreversion (Appendix 1—figure 23).

Let TP,FN,FP, and TN be, respectively, the number of true positives, false negatives, false positives and true negatives obtained from plasma donors and the pre-pandemic cohort in Manaus using a threshold 0.1. We use a uniform distribution in the interval [0, 1] as prior for the seroprevalence of age-sex group a, and also for the sensitivity se and specificity sp. The posterior distribution of the sensitivity and specificity is, respectively, $Beta\left(1+TP,1+FN\right)$ and $Beta\left(1+TN,1+FP\right)$. The seroprevalence ρ[a] of age-sex group a is distributed according to a binomial distribution of size T[a] (the number of tests for this age-sex group) and probability $se\times \rho \left[a\right]+(1-sp)(1-\rho \left[a\right])$. To draw a posterior sample of ρ[a], we draw a posterior sample of se and sp and from the auxiliary variable $Z[a]∼Beta(T^{+}\left[a\right]+1,T\left[a\right]-T^{+}\left[a\right]+1)$, which represents the raw measured prevalence. Then, we compute the prevalence adjusted by sensitivity and specificity through $\rho \left[a\right]=\left(0,\frac{Z\left[a\right]+\text{sp}-1}{\text{se}+\text{sp}-1}\right)\;.$ Finally, a sample of the IFR is then drawn from $Beta(1+D\left[a\right],1+\lfloor pop\left[a\right]\times \rho \left[a\right]\rfloor -D[a])$.

### Selection of SARI hospitalisations and deaths to estimate the IFR and IHR

In the first months of the SARS-CoV-2 epidemic in Brazil, a small proportion of SARI cases were tested for SARS-CoV-2, leading to a large number of non-notified deaths. For this reason, instead of using only COVID-19 confirmed hospitalisations or deaths to estimate the IFR and IHR, we also included SARI hospitalisations or deaths with unknown aetiology. This approach was proposed in de Souza et al., 2020. In this section, we investigate the validity of this approach by comparing SARI hospitalisations and deaths in the eight cities recorded between 2013 and 2021.

Appendix 1—figure 25 shows the monthly number of SARI deaths from 2013 to 2021 disaggregated by case classification (confirmed SARS-CoV-2 infection, infection confirmedly caused by other respiratory viruses, and cases with unknown or missing aetiology). The monthly number of SARI deaths increased abruptly in March 2020 due to the SARS-CoV-2 epidemic to 3810, 14.5 times larger than the previous peak of SARI cases in April 2016. Despite that only 58.6% of the SARI deaths in March 2020 were confirmed as COVID-19 cases, and 39.7% had unknown or missing aetiology, suggesting that most SARI deaths with unknown aetiology were non-notified COVID-19 deaths. Even if there was an epidemic of another respiratory virus in March 2020 that caused a number of cases similar to April 2016, it would only explain 17.4% of the SARI cases with unknown or missing aetiology in March 2020.

The proportion of each case classification among monthly SARI deaths is shown in Appendix 1—figure 4b. The proportion of deaths with unknown or missing aetiology had a peak in September 2020, decreasing over time in the following months likely due to the increasing availability of tests. Therefore, the effect of taking SARI into account is more important in the first year of the epidemic in Brazil.

Similar patterns are observed for SARI hospitalisations, as shown in Appendix 1—figure 26. However, SARI hospitalisations in March 2020 are only 4.7 times larger than the previous peak of monthly SARI hospitalisations in April 2016, suggesting that our approach is more sensitive to SARI cases caused by other respiratory viruses when hospitalisations are used. Nevertheless, an epidemic of other respiratory viruses similar to the historical peak of SARI cases in April 2016 would only explain 42% of the SARI cases with unknown or missing aetiology in March 2020.

### Method used to estimate $p^{+}[n]$ from repeat blood donors

Here we summarise the step-by-step procedure used to estimate $p^{+}[n]$, the probability of an individual remaining seropositive n weeks after seroconversion, described in Methods. The algorithm receives as inputs: The set of serial donations from $N_{donors}$ repeats blood donors who have at least one positive result and a second result with decaying S/C; the daily incidence over time for the repeat blood donors cohort $u_{repeat}[n]$ (see Methods); the number of samples $N_{samples}$ used to estimate the probability distribution of the time to seroreversion. The output of the algorithm is an estimate of $p^{+}[n]$.

The algorithm is described below:

- For $i\in 1,2,\cdots ,N_{donors}:$
  - Calculate the date of seroreversion for donor i by computing the instant where the exponential curve that passes through the last positive donation and first negative donation after seroconversion (if seroreversion occurred) or the two last positive donations cross the threshold, as illustrated in Appendix 1—figure 10. Denote it by $t_{i}^{-}.$
  - Denote $t_{min}$ as the last negative result of the donor before seroconversion or set $t_{min}$ as 1 March 2020, if the donor had no positive results before seroconversion. Denote $t_{max}$ as the date of the first positive result. The unobserved date of seroconversion belongs to the interval $\left[t_{min},t_{max}\right]$ and its probability mass function is given by
    $$p_{i}\left[n\right]=\left\{ \begin{array}{ll} \frac{u_{repeat}\left[n\right]}{\sum_{k=t_{min}}^{t_{max}}u_{repeat}\left[k\right]} & ,\;\;\;t_{min}\leq n\leq t_{max}\\ \;0 & ,\;\;\;\text{otherwise} \end{array}\right..$$
  - Generate $N_{samples}$ samples $\Delta t_{i}^{\left(1\right)},\Delta t_{i}^{\left(2\right)},\cdots ,\Delta t_{i}^{\left(N_{samples}\right)}$ from $\Delta t_{i}=t_{i}^{-}-t_{i}^{+}$, where $t_{i}^{+}∼p_{i}\left[n\right]$.
- Calculate the empirical probability mass function of $\Delta t_{i}$ by computing the empirical histogram of the generated samples $\Delta t_{i}^{\left(1\right)},\Delta t_{i}^{\left(2\right)},\cdots ,\Delta t_{i}^{\left(N_{samples}\right)}$ and denote it as $p_{day}^{-}[n]$.
- Convert $p_{day}^{-}[n]$ from days to weeks, obtaining $p_{week}^{-}[n]$:
  $$p_{week}^{-}\left[n\right]=\frac{1}{7}\sum_{i=1}^{7}\sum_{j=7n+1}^{7\left(n+1\right)}p_{day}^{-}\left[j-i\right].$$
- Calculate the probability of an individual remaining seropositive n weeks after seroconversion $p^{+}[n]$ as
  $$p^{+}\left[n\right]=1-\sum_{i=1}^{n}p_{week}^{-}\left[k\right].$$

### Description of the Bayesian model used to estimate the seroprevalence

We now present an objective description of the seroreversion correction model introduced in Methods. This is a Bayesian model that produces age- and sex-specific seroprevalence estimates corrected for seroreversion, sensitivity, and specificity. The model receives as inputs: The probability of seropositivity n weeks after seroconversion $p^{+}[n]$; the weekly number of tests T[n,a] and weekly number of positive tests $T^{+}\left[n,a\right]$ at week n for age-sex group a; the number of true positives (TP), true negatives (TN), false positives (FP) and false negatives (FN) used to determine the sensitivity and specificity of the assay; the maximum seroprevalence allowed b. In this work, we use b=2 to partially account for reinfections. The model generates as output posterior samples from the weekly incidence u[n,a] for age-sex group a=1,2,⋯,M and week n=1,2,⋯,N. For this reason, the model generates posterior samples from the sensitivity $se_{0},$ the specificity sp, the normalised incidence $u_{norm}[n,a]$, and the final seroprevalence $\rho_{max}[a]$, obtaining u[n,a] from these parameters.

The Bayesian model is described below:

- Prior distributions:
  $$se_{0}∼Beta(1+TP,1+FN)$$
  sp∼Beta(1+TN,1+FP)
  $$u_{norm}\left[1:N,a\right]∼Dirichlet\left(1,1,\cdots ,1\right)\forall a$$
  $$\rho_{max}\left[a\right]∼Uniform\left(0,b\right)\forall a$$
- Auxiliary variables:
  $$u\left[n,a\right]=u_{norm}\left[n,a\right]\times \rho_{max}\left[a\right],\forall n,a$$
  $$\theta \left[n,a\right]=se_{0}\sum_{k=1}^{n}p^{+}\left[n-k\right]u\left[k,a\right]+\left(1-sp\right)\left(1-\sum_{k=1}^{n}u\left[k,a\right]\right)\forall n,a$$
- Likelihood:
  $$T^{+}\left[n,a\right]∼Binomial\left(T\left[n,a\right],\theta \left[n,a\right]\right)\forall n,a$$

We note the estimated seroprevalence is the cumulative sum of the obtained incidence u[n,a] and can therefore be larger than 1 due to reinfections. Also, since this model assumes all infections occur in seronegative donors, u[n,a] can be interpreted as the incidence in seronegative donors, and reinfections among seropositive individuals are not detected.

### Summary of the method used to estimate the lower bound for the attack rate of the Gamma VOC in Manaus and the upper bound for the IFR

We now present a summary of the procedure used to infer bounds for the age-specific attack rate and IFR of the Gamma-dominated wave in Manaus explained in Methods. This procedure was also used to estimate the IHR, but in this case, the number of hospitalisations was used instead of the number of deaths.

The algorithm is executed independently for each age group and receives as inputs: The number of deaths D with symptom onset between 16 December 2020 and 15 March 2021; the monthly number of positive tests $T^{+}[n]$ and the monthly number of tests T[n] for $n\in \left\{12,13,14,15\right\}$ , i.e., from December 2020 (n=12) to March 2021 (n=15); the number of true positives (TP) and false negatives (FN) from the convalescent plasma donors cohort; population size pop.

The algorithm produces as outputs posterior samples of the maximum attack rate for the Gamma wave in Manaus (${AR}_{max}$) and the minimum IFR ($IFR_{min}$) but also generates posterior samples from the following auxiliary variables: The incidence between months n and n+1 denoted as u[n], and the seroprevalence in December 2020 (month n=12) denoted as $\rho_{December}$ .

First, we generate posterior samples from $\rho_{December}$ and u[n] using the Bayesian model described below:

- Prior distributions:
  $$\rho_{December}∼Uniform(0,1)$$
  $$\left(u_{norm}\left[12\right],u_{norm}\left[13\right],u_{norm}\left[14\right]\right)∼Dirichlet(1,1,1)$$
  $$u_{max}∼Uniform(0,1)$$
- Auxiliary variables:
  $$u\left[n\right]=u_{norm}\left[n\right]\times u_{max}\left[a\right],n\in \{12,13,14\}$$
- Likelihood:
  $$T^{+}\left[n,a\right]∼Binomial\left(T\left[n\right],\rho_{December}+\sum_{k=12}^{n-1}u\left[k\right]\right),n\in \left\{12,13,14,15\right\}$$

Then, for each posterior sample generated by the Bayesian model:

- Draw a sample from se∼Beta(1+TP,1+FN).
- Compute the incidence corrected by sensitivity $u^{corr}\left[n\right]=\frac{u\left[n\right]}{se}$ .
- Compute $\hat{AR}=\sum_{n=12}^{14}u^{corr}\left[n\right].$
- Compute $AR_{max}=\rho_{December}+\hat{AR}$ .
- Draw a sample from the lower bound of the IFR as
  $$IFR_{min}∼Beta\left(1+D,1+\left\lfloor AR_{max}\times pop\right\rfloor -D\right).$$

## References

[bib1] Banho CA, Sacchetto L, Campos G, Bittar C, Possebon FS, Ullmann LS, Marques B, da Silva GCD, Moraes MM, Parra MCP, Negri AF, Boldrin AC, Barcelos MD, dos Santos T, Milhim B, da Rocha LC, Dourado FS, dos Santos AL, Ciconi VB, Patuto C, Versiani AF, da Silva RA, Lobl E, Hernandes VM, Zini N, Pacca CC, Estofolete CF, Ferreira HL, Rahal P, Araújo JP, Cohen JA, Kerr CC, Althouse BM, Vasilakis N, Nogueira ML (2021). Effects of SARS-CoV-2 P.1 Introduction and the Impact of COVID-19 Vaccination on the Epidemiological Landscape of São José Do Rio Preto, Brazil. medRxiv.

[bib2] Barreto IC, Costa Filho RV, Ramos RF, Oliveira L de, Martins N, Cavalcante FV, Andrade L, Santos LMP (2021). Health collapse in manaus: the burden of not adhering to non-pharmacological measures to reduce the transmission of covid-19. Saúde Em Debate.

[bib3] Brazeau N, Verity R, Jenks S, Fu H, Whittaker C (2020). Report 34.

[bib4] Brizzi A, Whittaker C, Servo LMS, Hawryluk I, Prete CA, de Souza WM, Aguiar RS, Araujo LJT, Bastos LS, Blenkinsop A, Buss LF, Candido D, Castro MC, Costa SF, Croda J, de Souza Santos AA, Dye C, Flaxman S, Fonseca PLC, Geddes VEV, Gutierrez B, Lemey P, Levin AS, Mellan T, Bonfim DM, Miscouridou X, Mishra S, Monod M, Moreira FRR, Nelson B, Pereira RHM, Ranzani O, Schnekenberg RP, Semenova E, Sonnabend R, Souza RP, Xi X, Sabino EC, Faria NR, Bhatt S, Ratmann O (2021). Report 46: Factors Driving Extensive Spatial and Temporal Fluctuations in COVID-19 Fatality Rates in Brazilian Hospitals. medRxiv.

[bib5] Buss LF, Prete CA, Abrahim CMM, Mendrone A, Salomon T, de Almeida-Neto C, França RFO, Belotti MC, Carvalho M, Costa AG, Crispim MAE, Ferreira SC, Fraiji NA, Gurzenda S, Whittaker C, Kamaura LT, Takecian PL, da Silva Peixoto P, Oikawa MK, Nishiya AS, Rocha V, Salles NA, de Souza Santos AA, da Silva MA, Custer B, Parag KV, Barral-Netto M, Kraemer MUG, Pereira RHM, Pybus OG, Busch MP, Castro MC, Dye C, Nascimento VH, Faria NR, Sabino EC (2021). Three-quarters attack rate of SARS-cov-2 in the Brazilian Amazon during a largely unmitigated epidemic. Science.

[bib6] Buss LF, Sabino EC (2021). Intense SARS-cov-2 transmission among affluent manaus residents preceded the second wave of the epidemic in Brazil. The Lancet. Global Health.

[bib7] Castro MC, Kim S, Barberia L, Ribeiro AF, Gurzenda S, Ribeiro KB, Abbott E, Blossom J, Rache B, Singer BH (2021). Spatiotemporal pattern of COVID-19 spread in Brazil. Science.

[bib8] Coutinho RM, Marquitti FMD, Ferreira LS, Borges ME, da Silva RLP, Canton O, Portella TP, Poloni S, Franco C, Plucinski MM, Lessa FC, da Silva AAM, Kraenkel RA, de Sousa Mascena Veras MA, Prado PI (2021). Model-Based estimation of transmissibility and reinfection of SARS-cov-2 P.1 variant. Communications Medicine.

[bib9] Crotty S (2021). Hybrid immunity. Science.

[bib10] de Oliveira MHS, Lippi G, Henry BM (2021). Sudden Rise in COVID-19 Case Fatality among Young and Middle-Aged Adults in the South of Brazil after Identification of the Novel B.1.1.28.1 (P.1) SARS-CoV-2 Strain: Analysis of Data from the State of Parana. medRxiv.

[bib11] de Souza WM, Buss LF, Candido D, Carrera JP, Li S, Zarebski AE, Pereira RHM, Prete CA, de Souza-Santos AA, Parag KV, Belotti M, Vincenti-Gonzalez MF, Messina J, da Silva Sales FC, Andrade PDS, Nascimento VH, Ghilardi F, Abade L, Gutierrez B, Kraemer MUG, Braga CKV, Aguiar RS, Alexander N, Mayaud P, Brady OJ, Marcilio I, Gouveia N, Li G, Tami A, de Oliveira SB, Porto VBG, Ganem F, de Almeida WAF, Fantinato F, Macário EM, de Oliveira WK, Nogueira ML, Pybus OG, Wu CH, Croda J, Sabino EC, Faria NR (2020). Epidemiological and clinical characteristics of the COVID-19 epidemic in Brazil. Nature Human Behaviour.

[bib12] de Souza FSH, Hojo-Souza NS, da Silva CM, Guidoni DL (2021). Second wave of COVID-19 in Brazil: younger at higher risk. European Journal of Epidemiology.

[bib13] de Souza Santos AA, Candido D, de Souza WM, Buss L, Li SL, Pereira RHM, Wu CH, Sabino EC, Faria NR (2021). Dataset on SARS-cov-2 non-pharmaceutical interventions in Brazilian municipalities. Scientific Data.

[bib14] Di Germanio C, Simmons G, Kelly K, Martinelli R, Darst O, Azimpouran M, Stone M, Hazegh K, Grebe E, Zhang S, Ma P, Orzechowski M, Gomez JE, Livny J, Hung DT, Vassallo R, Busch MP, Dumont LJ (2021). SARS-cov-2 antibody persistence in COVID-19 convalescent plasma donors: dependency on assay format and applicability to serosurveillance. Transfusion.

[bib15] Faria NR, Mellan TA, Whittaker C, Claro IM, Candido D, Mishra S, Crispim MAE, Sales FCS, Hawryluk I, McCrone JT, Hulswit RJG, Franco LAM, Ramundo MS, de Jesus JG, Andrade PS, Coletti TM, Ferreira GM, Silva CAM, Manuli ER, Pereira RHM, Peixoto PS, Kraemer MUG, Gaburo N, Camilo C, Hoeltgebaum H, Souza WM, Rocha EC, de Souza LM, de Pinho MC, Araujo LJT, Malta FSV, de Lima AB, Silva J, Zauli DAG, Ferreira AC, Schnekenberg RP, Laydon DJ, Walker PGT, Schlüter HM, Dos Santos ALP, Vidal MS, Del Caro VS, Filho RMF, Dos Santos HM, Aguiar RS, Proença-Modena JL, Nelson B, Hay JA, Monod M, Miscouridou X, Coupland H, Sonabend R, Vollmer M, Gandy A, Prete CA, Nascimento VH, Suchard MA, Bowden TA, Pond SLK, Wu CH, Ratmann O, Ferguson NM, Dye C, Loman NJ, Lemey P, Rambaut A, Fraiji NA, Carvalho M, Pybus OG, Flaxman S, Bhatt S, Sabino EC (2021). Genomics and epidemiology of the P.1 SARS-cov-2 lineage in manaus, Brazil. Science.

[bib16] Hallal PC, Hartwig FP, Horta BL, Silveira MF, Struchiner CJ, Vidaletti LP, Neumann NA, Pellanda LC, Dellagostin OA, Burattini MN, Victora GD, Menezes AMB, Barros FC, Barros AJD, Victora CG (2020). SARS-cov-2 antibody prevalence in Brazil: results from two successive nationwide serological household surveys. The Lancet. Global Health.

[bib17] Lalwani P, Araujo-Castillo RV, Ganoza CA, Salgado BB, Jordão MF, Ortiz JV, Barbosa ARC, Sobrinho WBS, Cordeiro IB, Souza Neto JN, Assunção EN, Costa CF, Souza PE, Albuquerque BC, Astofi-Filho S (2021). High anti-SARS-cov-2 antibody seroconversion rates before the second wave in manaus, Brazil, and the protective effect of social behaviour measures: results from the prospective detectcov-19 cohort. The Lancet. Global Health.

[bib18] Li SL, Pereira RHM, Prete CA, Zarebski AE, Emanuel L, Alves PJH, Peixoto PS, Braga CKV, de Souza Santos AA, de Souza WM, Barbosa RJ, Buss LF, Mendrone A, de Almeida-Neto C, Ferreira SC, Salles NA, Marcilio I, Wu CH, Gouveia N, Nascimento VH, Sabino EC, Faria NR, Messina JP (2021). Higher risk of death from COVID-19 in low-income and non-white populations of São Paulo, Brazil. BMJ Global Health.

[bib19] Lucas C, Vogels CBF, Yildirim I, Rothman JE, Lu P, Monteiro V, Silva M, Tabachnikova J, Peña-Hernandez A, Muenker MA, Breban MC, Fauver MI, Mohanty S, Huang J (2021). Impact of circulating SARS-cov-2 variants on mRNA vaccine-induced immunity. Nature.

[bib20] Lumley SF, Wei J, O’Donnell D, Stoesser N, Matthews P, Howarth A, Hatch S, Marsden B, Cox S, James T, Peck L, Ritter T, Toledo Z, Cornall R, Jones EY, Stuart D, Screaton G, Ebner D, Hoosdally S, Crook D, Conlon C, Pouwels K, Walker A, Peto T, Walker TM, Jeffery K, Eyre D (2021). The duration, dynamics and determinants of SARS-cov-2 antibody responses in individual healthcare workers. Clinical Infectious Diseases: An Official Publication of the Infectious Diseases Society of America.

[bib21] Martins AF, Zavascki AP, Wink PL, Volpato FCZ, Monteiro FL, Rosset C, De-Paris F, Ramos ÁK, Barth AL (2021). Detection of SARS-cov-2 lineage P.1 in patients from a region with exponentially increasing hospitalisation rate, February 2021, Rio grande do sul, southern Brazil. Euro Surveillance.

[bib22] Mina MJ, Metcalf CJE, McDermott AB, Douek DC, Farrar J, Grenfell BT (2020). A global lmmunological observatory to meet a time of pandemics. eLife.

[bib23] Monod M, Blenkinsop A, Xi X, Hebert D, Bershan S, Tietze S, Baguelin M, Bradley VC, Chen Y, Coupland H, Filippi S, Ish-Horowicz J, McManus M, Mellan T, Gandy A, Hutchinson M, Unwin HJT, Elsland SL, Vollmer MAC, Weber S, Zhu H, Bezancon A, Ferguson NM, Mishra S, Flaxman S, Bhatt S, Ratmann O (2021). Age groups that sustain resurging COVID-19 epidemics in the United States. Science.

[bib24] O’Driscoll M, Ribeiro Dos Santos G, Wang L, Cummings DAT, Azman AS, Paireau J, Fontanet A, Cauchemez S, Salje H (2021). Age-Specific mortality and immunity patterns of SARS-cov-2. Nature.

[bib25] Orner EP, Rodgers MA, Hock K, Tang MS, Taylor R, Gardiner M, Olivo A, Fox A, Prostko J, Cloherty G, Farnsworth CW (2021). Comparison of SARS-cov-2 IgM and IgG seroconversion profiles among hospitalized patients in two us cities. Diagnostic Microbiology and Infectious Disease.

[bib26] Prete CA, Buss LF, Buccheri R, Abrahim CMM, Salomon T, Crispim MAE, Oikawa MK, Grebe E, da Costa AG, Fraiji NA, do P S S Carvalho M, Whittaker C, Alexander N, Faria NR, Dye C, Nascimento VH, Busch MP, Sabino EC (2022). Reinfection by the SARS-cov-2 gamma variant in blood donors in manaus, Brazil. BMC Infectious Diseases.

[bib27] Reynolds CJ, Pade C, Gibbons JM, Butler DK, Otter AD, Menacho K, Fontana M, Smit A, Sackville-West JE, Cutino-Moguel T, Maini MK, Chain B, Noursadeghi M, Network Uc, Brooks T, Semper A, Manisty C, Treibel TA, Moon JC, Investigators Uc, Valdes AM, McKnight Á, Altmann DM, Boyton R (2021). Prior SARS-cov-2 infection rescues B and T cell responses to variants after first vaccine dose. Science.

[bib28] Shioda K, Lau MSY, Kraay ANM, Nelson KN, Siegler AJ, Sullivan PS, Collins MH, Weitz JS, Lopman BA (2021). Estimating the cumulative incidence of SARS-cov-2 infection and the infection fatality ratio in light of waning antibodies. Epidemiology.

[bib29] Stamatatos L, Czartoski J, Wan YH, Homad LJ, Rubin V, Glantz H, Neradilek M, Seydoux E, Jennewein MF, MacCamy AJ, Feng J, Mize G, De Rosa SC, Finzi A, Lemos MP, Cohen KW, Moodie Z, McElrath MJ, McGuire AT (2021). Mrna vaccination boosts cross-variant neutralizing antibodies elicited by SARS-cov-2 infection. Science.

[bib30] Stone M, Grebe E, Sulaeman H, Di Germanio C, Dave H, Kelly K, Biggerstaff B, Crews BO, Tran N, Jerome KR, Denny TN, Hogema B, Destree M, Jones JM, Thornburg N, Simmons G, Krajden M, Kleinman S, Dumont LJ, Busch MP (2021). Evaluation of Commercially Available High-Throughput SARS-CoV-2 Serological Assays for Serosurveillance and Related Applications. medRxiv.

[bib31] Takahashi S, Greenhouse B, Rodríguez-Barraquer I (2020). Are seroprevalence estimates for severe acute respiratory syndrome coronavirus 2 biased?. The Journal of Infectious Diseases.

[bib32] Takahashi S, Peluso MJ, Hakim J, Turcios K, Janson O, Routledge I, Busch MP, Hoh R, Tai V, Kelly JD, Martin JN, Deeks SG, Henrich TJ, Greenhouse B, Rodríguez-Barraquer I (2021). SARS-CoV-2 Serology across Scales: A Framework for Unbiased Seroprevalence Estimation Incorporating Antibody Kinetics and Epidemic Recency. medRxiv.

