# Supplementary material for: SARS-CoV-2 antibody dynamics in blood donors and COVID-19 epidemiology in eight Brazilian state capitals: A serial cross-sectional study
Source: eLife. 2022 Sep 22;11:e78233. doi: 10.7554/eLife.78233 (PMC9545556; doi:10.7554/eLife.78233)
Supplement: Supplementary file 1. — Sensitivity and specificity were calculated by computing the quantiles of Beta (1+TP, 1+FN) and Beta (1+TN, 1+FP), respectively. [file elife-78233-supp1.docx]

|  | **Convalescent plasma donors** | | **Pre-pandemic blood donors cohort** | |  |  |
| --- | --- | --- | --- | --- | --- | --- |
| **Method (threshold)** | **Number of positive tests (TP)** | **Number of negative tests (FN)** | **Number of positive tests (FP)** | **Number of negatives tests (TN)** | **Sensitivity (%) and 95% CrI** | **Specificity (%) and 95% CrI** |
| **Anti-N assay; (1.4 S/C)** | 174 | 34 | 1 | 820 | 83.8 (78.0 - 88.4) | 99.8 (99.3, 100.0) |
| **Anti-N assay; (0.49 S/C)** | 189 | 19 | 20 | 801 | 90.6 (86.2, 94.0) | 97.5 (96.3 - 98.4) |
| **Anti-N assay; (0.1 S/C)** | 197 | 11 | 112 | 709 | 94.4 (90.8 - 97.0) | 86.3 (83.8 - 88.5) |
| **Anti-S assay; (50 S/C)** | 231 | 14 |  |  | 94.0 (90.6 - 96.5) | Assumed 100% |
