# Supplementary material for: SARS-CoV-2 antibody dynamics in blood donors and COVID-19 epidemiology in eight Brazilian state capitals: A serial cross-sectional study
Source: eLife. 2022 Sep 22;11:e78233. doi: 10.7554/eLife.78233 (PMC9545556; doi:10.7554/eLife.78233)
Supplement: Supplementary file 2. [file elife-78233-supp2.docx]

|  | **IFR obtained using the seroprevalence estimated with the proposed model (%)** | | | | | **IFR obtained using a threshold of 0.1 signal-to-cutoff to estimate the seroprevalence (%)** | | | | |
| --- | --- | --- | --- | --- | --- | --- | --- | --- | --- | --- |
| **Age group** | **16-24** | **25-34** | **35-44** | **45-54** | **55-64** | **16-24** | **25-34** | **35-44** | **45-54** | **55-64** |
| **Belo Horizonte** | 0.03 (0.02 - 0.05) | 0.09 (0.07 - 0.11) | 0.17 (0.14 - 0.21) | 0.46 (0.37 - 0.57) | 1.36 (0.99 - 1.96) | 0.05 (0.03 - 0.1) | 0.11 (0.07 - 0.18) | 0.26 (0.16 - 0.55) | 0.74 (0.44 - 1.43) | 2.38 (0.93 - 99.84) |
| **Curitiba** | 0.05 (0.03 - 0.07) | 0.08 (0.06 - 0.11) | 0.22 (0.17 - 0.27) | 0.74 (0.61 - 0.91) | 2.2 (1.6 - 3.16) | 0.05 (0.03 - 0.1) | 0.12 (0.07 - 0.22) | 0.34 (0.21 - 0.68) | 1.46 (0.74 - 6.31) | 4.16 (1.42 - 99.96) |
| **Fortaleza** | 0.03 (0.02 - 0.03) | 0.05 (0.04 - 0.06) | 0.16 (0.14 - 0.19) | 0.38 (0.33 - 0.45) | 0.9 (0.7 - 1.2) | 0.03 (0.02 - 0.04) | 0.06 (0.04 - 0.07) | 0.20 (0.16 - 0.27) | 0.42 (0.32 - 0.6) | 0.95 (0.65 - 1.99) |
| **Manaus** | 0.02 (0.01 - 0.02) | 0.04 (0.03 - 0.05) | 0.11 (0.1 - 0.13) | 0.31 (0.27 - 0.36) | 0.97 (0.8 - 1.19) | 0.02 (0.01 - 0.03) | 0.05 (0.04 - 0.06) | 0.15 (0.12 - 0.18) | 0.37 (0.3 - 0.47) | 1.21 (0.93 - 1.95) |
| **Recife** | 0.04 (0.03 - 0.05) | 0.09 (0.07 - 0.1) | 0.17 (0.15 - 0.2) | 0.48 (0.41 - 0.55) | 1.05 (0.85 - 1.33) | 0.05 (0.03 - 0.07) | 0.10 (0.08 - 0.13) | 0.2 (0.16 - 0.26) | 0.58 (0.44 - 0.81) | 1.3 (0.8 - 3.27) |
| **Rio de Janeiro** | 0.04 (0.03 - 0.04) | 0.08 (0.07 - 0.1) | 0.20 (0.18 - 0.23) | 0.46 (0.41 - 0.52) | 1.37 (1.16 - 1.65) | 0.04 (0.03 - 0.05) | 0.10 (0.08 - 0.12) | 0.24 (0.19 - 0.3) | 0.61 (0.47 - 0.82) | 2.13 (1.32 - 4.19) |
| **Salvador** | 0.02 (0.02 - 0.03) | 0.06 (0.05 - 0.08) | 0.16 (0.14 - 0.19) | 0.34 (0.29 - 0.41) | 0.97 (0.77 - 1.25) | 0.03 (0.02 - 0.05) | 0.09 (0.06 - 0.13) | 0.18 (0.14 - 0.24) | 0.49 (0.34 - 0.84) | 1.71 (1.04 - 3.43) |
| **São Paulo** | 0.04 (0.03 - 0.05) | 0.11 (0.09 - 0.12) | 0.20 (0.18 - 0.23) | 0.53 (0.46 - 0.63) | 1.65 (1.31 - 2.13) | 0.04 (0.03 - 0.05) | 0.14 (0.1 - 0.21) | 0.19 (0.15 - 0.25) | 0.53 (0.4 - 0.78) | 1.74 (0.97 - 5.23) |
