# Supplementary material for: SARS-CoV-2 antibody dynamics in blood donors and COVID-19 epidemiology in eight Brazilian state capitals: A serial cross-sectional study
Source: eLife. 2022 Sep 22;11:e78233. doi: 10.7554/eLife.78233 (PMC9545556; doi:10.7554/eLife.78233)
Supplement: Peer review history

# Editor report: Editor's evaluation

**DOI:** 10.7554/eLife.78233.sa0

**Authors:** James M McCaw

**Affiliations:** https://ror.org/01ej9dk98 The University of Melbourne Australia

This article describes a large and compelling COVID-19 sero-survey in Brazil that, when combined with death data, provides an estimate of the infection fatality ratio. This valuable study highlights both the strengths and challenges of blood donor sero-surveillance in a pandemic environment where multiple waves of infection occur and immune responses wane relatively quickly.

# Decision letter

**DOI:** 10.7554/eLife.78233.sa1

**Affiliations:** https://ror.org/01ej9dk98 The University of Melbourne Australia; https://ror.org/01b6kha49 Walter and Eliza Hall Institute of Medical Research Australia

In the interests of transparency, eLife publishes the most substantive revision requests and the accompanying author responses.

**Decision letter after peer review:**

Thank you for submitting your article "SARS-CoV-2 antibody dynamics in blood donors and COVID-19 epidemiology in eight Brazilian state capitals: A serial cross-sectional study" for consideration by *eLife*. Your article has been reviewed by 2 peer reviewers, and the evaluation has been overseen by a Reviewing Editor and Miles Davenport as the Senior Editor. The following individual involved in the review of your submission has agreed to reveal their identity: Ivo Mueller (Reviewer #1).

The reviewers have discussed their reviews with one another, and the Reviewing Editor has drafted this to help you prepare a revised submission.

In preparing a revised manuscript, please consider and address all comments raised by the reviewers. I draw your attention to one issue in particular though:

– The methodology uses deaths up to 15 Dec 2020 and compares that to the cumulative infections on 16 Dec 2020. As deaths lag infections, this will introduce a bias in the estimates of the IFR. While the bias is likely small due to the long study period and timing of waves of infection, in general, the approach should be adjusted to account for that lag.*Reviewer #1 (Recommendations for the authors):*

The authors conducted a retrospective sero-surveillance study of blood donor samples from the 8 largest cities in Brazil to determine the heterogeneity of age- and sex-specific exposure to SARS-CoV-2 infections and the resulting infection hospitalisation (IHR) and fatality rates (IFR). For Manaus, the observation period fully covered both primary and the Γ variant-of-concern wave allowing the investigators to compare IFRs between the two wave/viral variants.

The use of serology to track the COVID pandemic is complicated by the relatively rapidly waning antibody responses that lead to infected individuals' sero-reverting within 3-6 months after exposure. To adjust for that the authors developed an improved Bayesian seroreversion correction model that draws posterior samples from the incidence over time corrected by sensitivity, specificity, and sero-reversion rates drawn from repeat blood donor samples. This allowed them to not only undertake a robust and detailed investigation of SARS-CoV2 exposure patterns in Brazil and highlight the difference in the epidemic in different cities in Brazil but also to develop a methodology that can be applied for sero-surveillance of other pathogens.

This is a well-designed and executed study and very impressive in its scope and detail.

While the methods used for the sero-reversion estimation are not entirely novel but an extension of the approaches previously developed by Buss et al. 2021, their adjustments/addition are substantial and by deriving distribution from repeat blood donors and estimating serology-prevalence for each age group separately, their results will be more robust and accurate at least for infections acquired in the first wave.

As the authors outline very transparently, the estimations of attack/exposure rates in the 2nd/γ wave in Manaus are more difficult as their method does not allow identifying re-infection in already sero-positive individuals. Their method and assumption for estimating lower bounds for IFR and IHR for γ seem reasonable given the large difference in estimate IFR between Γ and 1st wave. This shortcoming may however become more pertinent if the same methods were used to calculate IFRs also for subsequent δ or omicron waves. While the authors do state the lower reliability of the inferences of attack rates towards the end of the time series, some more reflection on the circumstance/pandemic time frames that this approach can reliably be applied to might be useful.

Similarly, while blood donors are an easy source of blood samples they are a biased convenience sample of the overall population that the death and hospitalisations are drawn from. The authors show such biased for age, but other variables such as gender, socio-economic status, or general health status (and thus underlying health condition) may introduce further biases. While these may not change the overall time patterns of epidemic size, they might explain some of the smaller differences between cities or reasons. The authors are therefore very correct in stating that studies comparing blood donors with the general population are essential if blood donor surveillance is to become a standard tool for epidemic monitoring.

The heterogeneous patterns in attack rates are important for a more in-depth understanding of the pandemic course during a time and in locations where case ascertainment was low thus data on infection prevalence from diagnostic testing is very poor and difficult to interpret. Overall, they seem quite well in line with what was observed in other countries where overall case ascertainment was higher.

Despite its limitations, this is a very impressive and well-executed study that sets a high standard for other studies of sero-surveillance using blood donors.*Reviewer #2 (Recommendations for the authors):*

In the proposed paper, the authors use the results of antibody testing in samples from blood donations in different places in Brazil during the first year of the SARS-CoV-2 pandemic (2020). They develop models to account for antibody waning in estimating the cumulative seropositivity. They then use reported death data to infer the underlying IFR. This is a very large study, with an impressive amount of data. Understanding underlying differences in IFR across communities and the potential difference in IFR by variant remains of interest.

To estimate IFR, the authors count the number of deaths occurring prior to December 15, 2020, they then compare this to the proportion infected at December 16. This will lead to an underestimation, as the time from infection to death can be weeks – many individuals infected in mid-December 2020 who went on to die would not be included in the numerator. The authors could either use a later cut-off for the deaths or use deconvolution to estimate the total deaths attributable to seroprevalence at the cut-off date.

Furthermore, it would be useful to understand the potential for misclassification of deaths, as this is critical to the IFR estimates. The authors use SARI deaths rather than COVID-19-specific deaths. It would be good to understand what proportion of the SARI deaths were ultimately attributed to COVID-19 and how that changed over time. Maybe the authors could use the SARI deaths from the previous year as a reference for underlying non-COVID19 SARI?

Line 285. I was confused as to why a higher threshold limits the maximum number of reinfections? Is this as it would imply a higher antibody load and therefore a lower risk of re-infection.

Line 351. Is the 203 a 'mean' time between seroconversion and sero-reversion? Also, what are the units – days?

Two important age groups are not included in the blood bank data – those under 16y and those >64y. It was unclear to me how they were included in the overall IFR estimates (Figure 4).

Table 1. It was unclear to me what the differences between columns 2 and 3 are. Should column 3 maybe be titled 'Attack rate'?

In Figure 2 – the model results that adjust for seroreversion are lower than the crude estimates in pretty much each location for part of the study period. I would imagine that adjusting for seroreversion could only increase the seroprevalence.

Many of the paragraphs in the Results section are repetitions of the methods and could be removed. This will improve the readability of the paper.

Line 282 – what is S/C?

# Reply: Author response

**DOI:** 10.7554/eLife.78233.sa2

Reviewer #1 (Recommendations for the authors):

The authors conducted a retrospective sero-surveillance study of blood donor samples from the 8 largest cities in Brazil to determine the heterogeneity of age- and sex-specific exposure to SARS-CoV-2 infections and the resulting infection hospitalisation (IHR) and fatality rates (IFR). For Manaus, the observation period fully covered both primary and the Γ variant-of-concern wave allowing the investigators to compare IFRs between the two wave/viral variants.

The use of serology to track the COVID pandemic is complicated by the relatively rapidly waning antibody responses that lead to infected individuals' sero-reverting within 3-6 months after exposure. To adjust for that the authors developed an improved Bayesian seroreversion correction model that draws posterior samples from the incidence over time corrected by sensitivity, specificity, and sero-reversion rates drawn from repeat blood donor samples. This allowed them to not only undertake a robust and detailed investigation of SARS-CoV2 exposure patterns in Brazil and highlight the difference in the epidemic in different cities in Brazil but also to develop a methodology that can be applied for sero-surveillance of other pathogens.

We thank the reviewer for the kind remarks.

This is a well-designed and executed study and very impressive in its scope and detail.

While the methods used for the sero-reversion estimation are not entirely novel but an extension of the approaches previously developed by Buss et al. 2021, their adjustments/addition are substantial and by deriving distribution from repeat blood donors and estimating serology-prevalence for each age group separately, their results will be more robust and accurate at least for infections acquired in the first wave.

As the authors outline very transparently, the estimations of attack/exposure rates in the 2nd/γ wave in Manaus are more difficult as their method does not allow identifying re-infection in already sero-positive individuals. Their method and assumption for estimating lower bounds for IFR and IHR for γ seem reasonable given the large difference in estimate IFR between Γ and 1st wave. This shortcoming may however become more pertinent if the same methods were used to calculate IFRs also for subsequent δ or omicron waves. While the authors do state the lower reliability of the inferences of attack rates towards the end of the time series, some more reflection on the circumstance/pandemic time frames that this approach can reliably be applied to might be useful.

Similarly, while blood donors are an easy source of blood samples they are a biased convenience sample of the overall population that the death and hospitalisations are drawn from. The authors show such biased for age, but other variables such as gender, socio-economic status, or general health status (and thus underlying health condition) may introduce further biases. While these may not change the overall time patterns of epidemic size, they might explain some of the smaller differences between cities or reasons. The authors are therefore very correct in stating that studies comparing blood donors with the general population are essential if blood donor surveillance is to become a standard tool for epidemic monitoring.

The heterogeneous patterns in attack rates are important for a more in-depth understanding of the pandemic course during a time and in locations where case ascertainment was low thus data on infection prevalence from diagnostic testing is very poor and difficult to interpret. Overall, they seem quite well in line with what was observed in other countries where overall case ascertainment was higher.

Despite its limitations, this is a very impressive and well-executed study that sets a high standard for other studies of sero-surveillance using blood donors.

We appreciate your suggestions. Our method can be applied to Δ and Omicron waves as long as the initial crude seroprevalence is small. In addition, it cannot distinguish seroconversion due to vaccination or natural infection. As such, it is more interesting to use our approach in regions or time-periods where vaccines that induce anti-nucleocapsid antibodies were not frequently administered. We added the sentence below in line 350 to discuss the reliability of our approach to estimate the attack rate of epidemics with non-negligible rates of reinfection.

“This method can be applied to estimate upper bounds for the attack rate of epidemics driven by other lineages with high rates of reinfection such as Delta and Omicron VOCs, but as previously highlighted the upper bound is only informative if the initial crude seroprevalence is small. This may not be the case in regions where vaccines inducing anti-nucleocapsid antibodies were applied, as it is not possible to distinguish vaccination from natural infection based only on anti-N serological data.”

We also added a sentence in line 285 to emphasize the increasing uncertainty of the seroprevalence and IFR estimates over time:

“We note that the proposed seroreversion correction model can be used to estimate the attack rate and IFR of epidemics driven by other lineages in other regions. However, the uncertainty of the seroprevalence estimate increases over time, as a larger amount of seroreversion needs to be corrected. Therefore, estimated attack rates and IFRs suffer from larger uncertainty when longer time periods are considered.”

Reviewer #2 (Recommendations for the authors):

In the proposed paper, the authors use the results of antibody testing in samples from blood donations in different places in Brazil during the first year of the SARS-CoV-2 pandemic (2020). They develop models to account for antibody waning in estimating the cumulative seropositivity. They then use reported death data to infer the underlying IFR. This is a very large study, with an impressive amount of data. Understanding underlying differences in IFR across communities and the potential difference in IFR by variant remains of interest.

To estimate IFR, the authors count the number of deaths occurring prior to December 15, 2020, they then compare this to the proportion infected at December 16. This will lead to an underestimation, as the time from infection to death can be weeks – many individuals infected in mid-December 2020 who went on to die would not be included in the numerator. The authors could either use a later cut-off for the deaths or use deconvolution to estimate the total deaths attributable to seroprevalence at the cut-off date.

Furthermore, it would be useful to understand the potential for misclassification of deaths, as this is critical to the IFR estimates. The authors use SARI deaths rather than COVID-19-specific deaths. It would be good to understand what proportion of the SARI deaths were ultimately attributed to COVID-19 and how that changed over time. Maybe the authors could use the SARI deaths from the previous year as a reference for underlying non-COVID19 SARI?

The SIVEP-Gripe dataset contains not only the date of death for each deceased patient, but also the date of first symptoms. As such, to estimate the IFR for 2020 we use deaths from patients with symptom onset before December 15, instead of using deaths that occurred before December 15. For this reason, it is not necessary to take into account the delay between deaths and symptoms onset. We apologize that this information is not clear in the manuscript.

This information was mentioned in line 263:

“To estimate the IFR in 2020, we use the seroprevalence estimated by our model for December 16, 2020 and select only SARI deaths with symptoms onset between March 1st and December 15, 2020.”

To clarify this sentence, we added a second sentence after it:

“Selecting deaths based on the date of first symptoms instead of date of death was possible because SIVEP-Gripe contains the date of symptom onset for each individual.”

We also emphasized we used the date of symptom onset in the legend of Figure 4:

“The number of deaths was obtained from the SIVEP-Gripe reporting system including all SARI deaths with symptom onset between March 1^st^, 2020 and December 15^th^, 2021.”

We appreciate your suggestion to analyze the potential impact of misclassification of deaths. We included a reference in line 261 for the first paper that uses SARI deaths as a proxy for COVID-19 deaths. We also added a supplementary analysis in Appendix 1 (line 960), showing that it is reasonable to assume the large majority of unspecified SARI deaths after March 2020 are undetected COVID-19 deaths. Furthermore, as explained in the new supplementary analysis, the proportion of SARI deaths with unknown etiology decays with time as tests become more available. Therefore, the monthly number of imputed COVID-19 deaths based on unspecified SARI deaths decreases with time, thus the effect of imputation in the IFR estimate is more relevant during the first months of the epidemic.

Line 285. I was confused as to why a higher threshold limits the maximum number of reinfections? Is this as it would imply a higher antibody load and therefore a lower risk of re-infection.

Using a higher threshold lowers the number of seropositive donors in December 2020, limiting the number of estimated reinfections among seropositive donors and leading to a smaller estimate of the upper bound for the attack rate.

We believe the sentence in Line 285 of the original manuscript is not clear enough. Therefore, we rewrote this sentence, and added two paragraphs in Lines 314 and 336 of the revised version of the manuscript to improve the explanation of our method. We hope this modification clarifies the reason why we use a larger threshold of 1.4 to estimate the attack rate of the second wave in Manaus. We also improved the description of our method in Appendix 1 – Algorithm 3 (moved to the main text in Appendix 1).

“We first estimate the attack rate of the second wave using a Bayesian model that does not take reinfections into account. This model also neglects seroreversion for individuals infected during the second wave due to the small interval of three months considered in this analysis (see Appendix 1 for a complete description of the model). Denoting as $\hat{\text{AR}}$ the attack rate estimated by this model, the true attack rate AR is given by $\text{AR}=\hat{\text{AR}}+R+S$, where R is the proportion of donors that were seropositive in December 2020 and subsequently had a reinfection, and S is the proportion of donors that were seropositive in December 2020 and became seronegative in the following months. Since R+S cannot be greater than the seroprevalence in December 2020 (denoted as $\rho_{December}$), the upper bound for the attack rate is ${\text{AR}}_{\max}=\hat{\text{AR}}+\rho_{\text{December}}$. Therefore, the upper bound is obtained assuming that all individuals that were seropositive in December were later reinfected or were seronegative in March 2021.

To estimate ${\text{AR}}_{\max}$, we compute the monthly number of positive tests $T^{+}\left[n\right]$ from December 2020 to March 2021 for each age-sex group, as well as the number of True Positive (TP) and False Negatives (FN) from convalescent plasma donors and the number of False Positives (FP) and True Negatives (TN) from the pre-pandemic blood donors cohort in Manaus (Supplementary File 1). The Bayesian model generates posterior samples of the crude monthly incidence and the crude seroprevalence in December $\rho_{December}$. We then correct the crude incidence by the sensitivity of the assay to obtain posterior samples of $\hat{AR}$, which are then added to the posterior samples of $\rho_{\text{December}}$, resulting in samples of ${\text{AR}}_{\text{max}}$. As explained above, the lower bound for the IFR is then calculated using the upper bound of the attack rate and the number of deaths with symptom onset between December 16 and March 15. This procedure is repeated for each age-sex group independently, and is summarised in Appendix 1.

Only small estimates of the upper bound for the attack rate are informative, as in scenarios where $\rho_{\text{December}}$ is small. To limit $\rho_{\text{December}}$, we estimate the incidence using a threshold of 1.4 signal-to-cutoff (the upper threshold recommended by the manufacturer) instead of 0.49 signal-to-cutoff (the lower threshold recommended by the manufacturer), and correct for sensitivity based on 163 true positives and 30 false negatives in the plasma donors cohort. Since the specificity of the test using a threshold of 1.4 is 99.9%, and since it is not straightforward to take the specificity into account when reinfections are allowed, we do not correct for specificity in this analysis.”

Line 351. Is the 203 a 'mean' time between seroconversion and sero-reversion? Also, what are the units – days?

203 and 280 are respectively the median time between seroconversion and seroreversion for repeat blood donors and convalescent plasma donors, measured in days. We changed the sentence to:

“yielding a shorter median time between seroconversion and seroreversion (203 [147 – 294] days *versus* 280 [175 – 441] days)”.

Two important age groups are not included in the blood bank data – those under 16y and those >64y. It was unclear to me how they were included in the overall IFR estimates (Figure 4).

To facilitate the comparison of the IFRs across cities, we combined the age-specific IFR estimates to obtain an overall IFR for individuals aged between 16 and 64 instead of an overall IFR that includes all age groups. As such, the IFR of individuals older than 64 or younger than 16 is not included in this estimate. We highlighted in the legend of Figure 4 that overall IFR estimates consider only the age range 16-64, and added a paragraph in line 279 to clarify this information:

“To infer the IFR, we considered the age groups 16-24, 25-34, 35-44, 45,54 and 55-64. We applied the same method to estimate the overall IFR but using a single age group containing all individuals aged between 16 and 64. Therefore, IFR of individuals older than 64 or younger than 16 are not included in the overall IFR estimates. The method used to infer the IFR was also applied to compute the Infection-Hospitalisation Rate (IHR), but we used the number of hospitalisations with SARI instead of the number of deaths.”

Table 1. It was unclear to me what the differences between columns 2 and 3 are. Should column 3 maybe be titled 'Attack rate'?

Column 3 is the estimated attack rate, obtaining by correcting the crude seroprevalence by sensitivity, specificity, seroreversion and reweighting by age and sex. Column 2 is the seroprevalence corrected by sensitivity, specificity and reweighted by age and sex, but not corrected for seroreversion.

We agree that ‘Attack Rate’ is a better name for the 3rd column. We also changed the name of the 2nd column to “Adjusted seroprevalence with no correction for seroreversion (%)” to clarify its meaning.

In Figure 2 – the model results that adjust for seroreversion are lower than the crude estimates in pretty much each location for part of the study period. I would imagine that adjusting for seroreversion could only increase the seroprevalence.

Figure 2 shows the adjusted seroprevalence obtained by aggregating all age and sex groups, but the model produces age- and sex- specific seroprevalence estimates. Appendix 1 – Figure 16 compares the age- and sex- specific crude seroprevalence with the estimates from our model. In almost all locations, months and age-sex groups, the estimated seroprevalence lie within or above the 95% confidence intervals of the crude seroprevalence.

However, in some cases the age- and sex- specific seroprevalence corrected for seroreversion is smaller than the crude seroprevalence. This would not be possible in deterministic models that fit exactly the measured seroprevalence such as in Buss et al., but it may happen in Bayesian models in time points where the crude seroprevalence does not agree with the model. Therefore, our Bayesian model not only corrects for seroreversion, but also partially removes inconsistent samples. This is because seroprevalence curves that cannot be reconstructed by the model generate a smaller likelihood, hence a smaller probability of being included in the set of samples generated by the model.

An inconsistent sample may occur in weeks where blood donors are not representative of the population, being biased towards seropositive or seronegative individuals, or due to sampling noise. This effect is discussed for Fortaleza in Line 466:

“The estimated seroprevalence in June and July in Fortaleza was significantly smaller than the measured seroprevalence without correction for seroreversion, even though the seroprevalence estimates disaggregated by age and sex (Appendix 1 – Figure 16) lie within or above the confidence intervals of the measured seroprevalence. This effect happened especially in women, which had a crude seroprevalence that was significantly larger than in men in June and July 2020, but became similar in the following months. It is possible that the seroreversion rate observed in Fortaleza had been faster than the rate estimated from repeat blood donors, in which case we undercorrected for seroreversion, underestimating the attack rate. However, a more likely explanation is that samples between March and July 2020 for Fortaleza are less representative of the population, since only 39.4% from 4,970 selected samples could have been retrieved and tested, compared to 97.0% for the other cities and months. As such, seropositive individuals from Fortaleza may have been more likely to donate in these months, leading to an overestimated crude seroprevalence.”

To better explain this effect, we added a paragraph in line 236:

“It is worth noting that the age-specific crude seroprevalence can be larger than the seroprevalence corrected for seroreversion in some weeks, as the model may remove outlier samples. This is because seroprevalence curves that cannot be reconstructed by the model (for example, due to bias or sampling noise) generate a small likelihood, hence a smaller probability of being included in the set of posterior samples generated by the model. Therefore, the model excludes weeks where donors are significantly biased towards more seropositive or more seronegative individuals.”

Many of the paragraphs in the Results section are repetitions of the methods and could be removed. This will improve the readability of the paper.

We appreciate your suggestion. We moved some of the paragraphs in the Results section to Methods and removed sentences that contained repeated information.

Line 282 – what is S/C?

S/C is an abbreviation for signal-to-cutoff, which is the analog result of the test. If the signal-to-cutoff is greater than or equal to the predefined threshold, the test result is positive. We added a sentence in line 89 to clarify the meaning of the abbreviation S/C:

“A test is considered positive if the obtained signal-to-cutoff (S/C) is greater or equal to a predefined threshold of 0.49.”
